# Supplementary material for: Carbon footprint distributions of lithium-ion batteries and their materials
Source: Nat Commun. 2024 Nov 27;15:10301. doi: 10.1038/s41467-024-54634-y (PMC11603021; doi:10.1038/s41467-024-54634-y)
Supplement: Supplementary file 1 — Supplementary Information [file 41467_2024_54634_MOESM1_ESM.pdf]

Supplementary Information

for the publication

Carbon Footprint Distributions of
Lithium-Ion Batteries and Their Materials

Leopold Peiseler[1,2,3*], Vanessa Schenker[4], Karin Schatzmann[1,3], Stephan Pfister[3,4], Vanessa Wood[2,3], Tobias Schmidt[1,3]

[1] Energy and Technology Policy Group, ETH Zurich; Clausiusstrasse 37, CH-8092 Zurich, Switzerland

[2] Materials and Device Engineering Group, ETH Zurich; Gloriastrasse 35, CH-8092 Zurich, Switzerland

[3] Institute of Science, Technology and Policy, ETH Zurich; Universitätstrasse 41, CH-8092 Zurich, Switzerland

[4] Chair of Ecological Systems Design, ETH Zurich; Laura-Hezner-Weg 7, CH-8093 Zurich, Switzerland

*corresponding author: pleopold@ethz.ch

Table of References

Supplementary Methods 4
Supplementary Note 1 Breakdown of CF contributions 13
Supplementary Note 2 Additional emission curves 20
Supplementary Note 3 Additional CF distributions 22
Supplementary Note 4 Extensive literature review of battery material CFs 26
Supplementary References 37

List of Supplementary Figures

Supplementary Figure 1: Modelling workflows.. 6
Supplementary Figure 2: Probability density functions based on emission curves.. 9
Supplementary Figure 3: Emission curves, literature and database carbon footprint values and 2022 supply chain for graphite. 20
Supplementary Figure 4: Emission curves, literature and database carbon footprint values and 2022 supply chain for nickel sulfate equivalent, excluding laterite ore deposits. 21
Supplementary Figure 5: Probability density distribution and jitter plot for LIB cells with NMC811 cathodes, including laterite ore deposits in per-kg units. 22
Supplementary Figure 6: Probability density distribution and jitter plot for LIB cells with LFP cathodes in per kg units.. 22
Supplementary Figure 7: Carbon footprint profile of battery cells with NMC811 cathodes, excluding laterite ore deposits. 23
Supplementary Figure 8: Carbon footprint profile of battery cells with LFP cathodes synthesised via the solid-state method.. 24
Supplementary Figure 9: Carbon footprint profile of battery cells with LFP cathodes synthesised via the hydrothermal method.. 25

List of Supplementary Tables

Supplementary Table 1: Uncertainty distributions of input variables for emission modelling of S&P Global's output products.. 8
Supplementary Table 2: List of expert interviews conducted in the context of this study 11
Supplementary Table 3: Statistical values (5th, 50th, 95th percentiles, standard deviation and variance) for datasets underlying Figures 2 and 3 and Supplementary Figures 7,8, and 9. ..12
Supplementary Table 4: Disaggregated relative and absolute CF contributions of exchanges and activities for the production of NMC811 battery cells (including laterite). 13
Supplementary Table 5: Disaggregated relative and absolute CF contributions of exchanges and activities for the production of LFP battery cells (90% solid-state share). 16
Supplementary Table 6: Tabulated variance breakdown for all chemistries and variations...20
Supplementary Table 7: Search metadata for lithium literature review. 28
Supplementary Table 8: Search metadata for nickel literature review. 30
Supplementary Table 9: Search metadata for cobalt literature review. 33
Supplementary Table 10: Search metadata for graphite literature review. 35

Supplementary Methods

General S&P Global modelling remarks

S&P Global's Capital IQ Pro offers a tool called Mine Economics,[1] an effort to model mine-level costs for various commodities, including lithium, cobalt and nickel. The rest of this section summarises the methodological details pertinent to this work and is entirely based on S&P Global's methodological description.[2] For further questions regarding the modelling decisions of S&P Global, contact the corresponding author.

The S&P Global team uses a bottom-up approach to estimate mining and ore processing costs, focusing on the required process flowsheet, equipment and operational workforce. This includes assessing consumable costs. Analyses typically begin with a technical, feasibility or pre-feasibility report on the asset and other related corporate disclosures, such as presentations, financial reports and conference call transcripts. S&P Global's analysts bolster this data with direct communications with the owning company and site visits. When detailed cost breakdowns are lacking, they apply industry benchmarks or calculations for factors such as reagent consumption and electricity usage. All modelled figures are aligned with available reported disclosures, and a reconciliation is provided on the notes page for each model.

Analysts initiate their cost modelling with a detailed mine plan, drawing from technical reports and historical data. They model the process flowsheet (i.e. individual processing steps), including ore and waste handling, processing methodologies and the requisite labour and equipment. This granular approach extends to assessing input costs, including consumables such as fuel and chemicals, as well as labour and energy costs, to accurately determine the cost per tonne of ore processed.

The team then aligns these modelled estimates with actual operational and financial data from companies, refining their models to ensure that they reflect real-world operations. This results in a granular breakdown of costs into categories, including labour, energy, fuel and reagents, among others, closely mirroring company disclosures. This blend of bottom-up and top-down analysis offers a nuanced view of mining and milling expenses.

Treatment and refining Charges (TC/RCs) for intermediate products, such as concentrates, are based on global benchmarks tailored for specific impurities and concentrate grades and set within the framework of annual or multi-year agreements. S&P Global bases these benchmarks on its own market intelligence and external sources, such as S&P Global Platts, to ensure that the modelled TC/RCs remain relevant and accurate over time, providing a stable basis for future projections. Finally, cost data are provided on a per-metal-paid basis, accounting for varying product types and grades. For this study, S&P Global provided costs for lithium in lithium carbonate equivalent (LCE) units, but they were stoichiometrically converted by the authors to per-metal-paid for consistency with other metals. Following correspondence with S&P Global analysts, all mining costs on a per-metal-paid basis (i.e. costs for nickel and cobalt mines) were normalised by their payability to incorporate mining costs for ore that was indeed extracted but not refined to a usable product.

Preparing S&P Global data

By- vs. co-product

For assets with multiple mining outputs, S&P Global provides two different allocation approaches: by-product and co-product. These methods offer different means of distributing the total cost among the metals produced.

In by-product accounting, all shared mining, milling and processing costs are attributed to the primary metal, while costs specific to each metal, such as refining costs, are allocated

accordingly. For instance, in the case of a mine that primarily produces copper but also cobalt as a sellable side product, all shared costs are attributed to copper products. Shared costs refer to processes that treat both copper and cobalt, such as ore extraction or flotation. Refining charges for by-products are subtracted from their gross revenue to calculate the by-product credit, which is then deducted from the primary metal's cash operating costs.

Co-product accounting, on the other hand, divides costs based on the revenue contribution of each metal in the intermediate product. After refining charges are subtracted from each metal's revenue, treatment and freight charges are allocated proportionally to the contained metals based on their share of net revenue. For example, if copper accounts for 76% of net revenue, it will bear a corresponding percentage of shared production costs.

For this study, costs based on co-product allocation were chosen. This decision was made because it aligns well with the economic allocation approach of the life cycle assessment (LCA) literature, whereas "by-product" does not conceptually match the LCA mass-based allocation method. With the "by-product" approach, cobalt, which is typically mined as a secondary metal, would be underrepresented in the majority of costs and thus emissions.

Exclusion of assets

As explained in the method section of the main text, the technical fields of all assets (e.g. geology, flowsheet properties, output products and modelling choices) were manually checked and harmonised for plausibility and consistency. In the next step, for every commodity, assets were grouped into output products (lithium: lithium carbonate and lithium concentrate; nickel: nickel concentrate; cobalt: cobalt hydroxide and cobalt concentrate). Assets with multiple or unclear output products were excluded from the short list. Furthermore, assets with inconsistent flowsheet cost information were removed. Nickel assets producing exclusively ferronickel were also excluded since there is no commercially established practice for converting ferronickel into nickel sulfate. See the section "Building global battery-specific supply chains" of this document for nickel-specific supply chain modelling choices.

Selection of cost categories

For every asset, S&P Global provides Total Production Costs that are composed of All-in-Costs plus Reclamation, Depreciation, and Inventory Changes, among others (the capitalisation of cost categories in this study followed S&P Global nomenclature). All-in-Costs, in turn, are the sum of Cash Operating Costs, Royalties and Taxes, Corporate Overhead, Sustaining and Expansion Capex, and Interest Charges. These cost categories are not directly tied to operating greenhouse gas emission (GHG) emissions. However, Cash Operating Costs are composed of Offsite Transport costs, TC/RCs, and Total Minesite Costs, which, in turn, comprise Labour, Fuel, Electricity, Reagent, and Other costs for the mine and mill stages.

The methodology proposed in this work leveraged offsite transport costs and the Minesite Cost components – Labour, Fuel, Electricity, and Reagent – to estimate emissions at the mine and mill stages. Additionally, TC/RCs were considered to have a signalling effect for refining emissions (see below). "Other" mill and mine costs encompassed blasting and geologic consulting costs in varying proportions (and thus with varying GHG implications); this explains why this cost category could not be used to determine emissions. Furthermore, royalties, labour and other sustaining costs do not carry meaningful information for deducing emissions.

TC/RCs are fees charged by smelters for processing intermediate metal products, with rates influenced by global benchmarks, the presence of impurities and the grade of the concentrate. These charges can vary based on contract terms and are often negotiated based on specific supply conditions, such as the quantity and quality of the metal being processed.[2] Due to the fluctuating and market-dependent nature of TC/RCs, they could not be directly included in the

bottom-up emission model. However, acknowledging their relevance in relation to GHG emissions, TC/RCs were utilised as indicators to approximate the most likely positioning of assets along the refining emissions spectrum. As a conservative modelling approach, we maintained the full range of uncertainty for refining emissions based on values from the literature, but applied TC/RCs to determine the most probable emission values.

Further information on the emission modelling of output products

Supplementary Figure 1 graphically illustrates the modelling steps from S&P Global cost data to emissions using various data sources. This figure corresponds to equations (1)–(8) in the main text's method section.

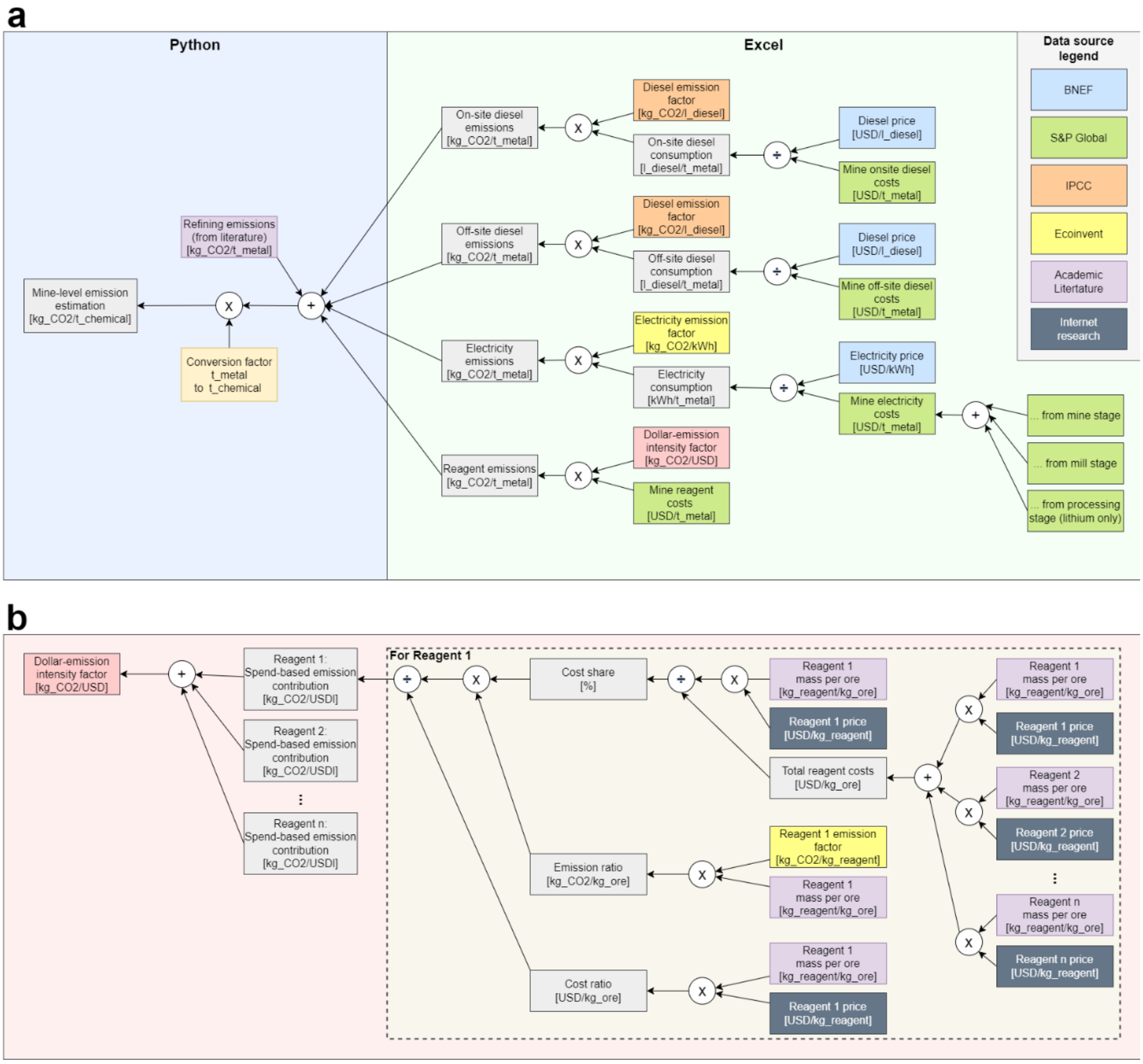

*Supplementary Figure 1: **Modelling workflows. a** Modelling workflow for emission calculations in Excel and Python. **b** Detailed workflow for determining the spend-based emission factors for reagent emission modelling.*

Note that for four nickel assets in South Africa, the dense media separation (DMS) process step was omitted, with only flotation considered. Given that these were the only assets necessitating DMS modelling and that their combined production share was minimal (less than 0.08%), it was determined that the extra effort required for modelling DMS was unwarranted.

Modelling on-site electricity production

While most assets are physically connected to the grid, other remote assets produce their electricity on-site. Naturally, the differentiation between grid and on-site electricity production was crucial for emission model accuracy. Thus, based on S&P Global's modelling notes, we explicitly calculated electricity emissions for on-site production through the diesel and natural gas (NG) routes. As for on-site and off-site transportation, diesel prices and emissions were taken from Bloomberg New Energy Finance (BNEF)[3] and Intergovernmental Panel on Climate Change (IPCC)[4] respectively. For the two countries where NG is used for electricity production (Argentina and Australia), NG prices were retrieved from internet research. Sources and modelling details can be found in the sheet "Natural Gas Prices 2022 (MC)" in "SP_CIQ_Lookup_File.xlsx".

It should be noted that the emission factors for both diesel and NG were treated without regional differentiation because the variations across regions were considered negligible, as indicated by the IPCC (see Tables 1.4 and 1.6[4]). Additionally, for diesel, no distinction was made between stationary and mobile combustion sources, as the discrepancy in values was minimal, with an error of less than 0.1% (see Tables 1.5 and 1.6[4]).

Sources and production volumes for graphite and nickel laterite

Since only carbon footprint (CF) data for Chinese graphite production were available, only Chinese natural and artificial production volumes were of interest. The production volumes of Chinese graphite were taken from the previously developed battery-specific graphite supply chain. The same approach was applied to determine the nickel laterite production volumes for Indonesia, the Philippines and New Caledonia (sui generis collectivity of France). These were the only countries of interest, as they were the only ones for which CF estimates were available. The sources providing nickel sulfate CF estimates from laterite mining broke down Indonesian operations into limonite processing via high-pressure acid leaching (HPAL) and saprolite via rotary kiln electric furnace (RKEF) mining. Both limonite and saprolite are subcategories of laterite deposits.

To make use of this differentiation, it was necessary to determine the share of saprolite in laterite mining in Indonesia. To this end, we turned to internet research, as the industrial processing of saprolite via nickel pig iron (NPG) and nickel matte to nickel sulfate using RKEF processes is not well established and is currently only carried out by China's Tsingshan Holding Group[5] Typical process flowsheets for sulfide and laterite (limonite and saprolite) can be found in two of the references of the present study.[5,6] Tsingshan announced that in 2021, the firm planned to produce 100 kt of nickel matte that year[7] As this is the only available figure, we use the nickel content in matte (ca. 75%[8]) and nickel sulfate (22%, stoichiometrically determined) and convert it to 341 kt of nickel sulfate derived from saprolite. We assume that the rest of Indonesia's laterite supply and all that of the Philippines and New Caledonia stem from limonite ore and are processed using HPAL.

Input distributions and uncertainties for material Monte Carlo simulation

To conduct uncertainty modelling of the output products' emissions, the uncertainty distributions of the input parameters (cf. Supplementary Figure 1) had to be defined. As explained in the main text's method section, "Uncertainty quantification – Material Monte Carlo simulation", the Excel plugin @risk was used for operationalisation. The following input uncertainty distributions (see Supplementary Table 1) were chosen:

Supplementary Table 1: Uncertainty distributions of input variables for emission modelling of S&P Global's output products. These fields correspond to the input fields in Supplementary Figure 1.

Input variable	Value	Distribution type	Comment/Source
S&P Global cost data	+− 10 %	Triangular	Generic uncertainty for S&P Global data (based on expert interview with data provider)
Diesel prices	+− 5 %	Triangular	Generic uncertainty for BNEF data
Natural gas (NG) prices	NA	Uniform	Sampled across all monthly 2022 country-specific prices and FX rates retrieved from the internet
Reagent prices	NA	Uniform	If sources provide range
	NA	Triangular	If there are multiple values from multiple sources
	+− 10%	Triangular	Generic uncertainty if only a singular value is available
Diesel/NG carbon footprint (CF)	NA	NA	No uncertainty assumed based on IPCC work[4]
Electricity grid CF	NA	Uniform	Based on 600 Monte Carlo samples of "market [group] for electricity, high voltage" [kg_{CO2} kWh^{-1}] for every country/region using IPCC2021 no LT GWP100 LCIA method and ecoinvent 3.9.1
Reagent CF	NA	Uniform	Based on 600 Monte Carlo samples of the ecoinvent chemical activity [kg_{CO2} kg^{-1}] for every reagent using IPCC2021 no LT GWP100 LCIA method and ecoinvent 3.9.1

Aggregating modelled and refining emissions and margins of error

The general approach to adding refining emissions and margins of error is described in the main method section. Specifically, for cobalt sulfate derived from cobalt hydroxide, it should be noted that there was no existing work meeting the requirements (mainly no breakdown along processing steps) outlined in the main method section "Comparing modelled emissions to literature and database values" and Supplementary Note 4. Given this lack of existing literature, the ecoinvent 3.9.1 activity for converting cobalt hydroxide to cobalt sulfate was used. First, the unit CFs of all exchanges of this activity were separately calculated 500 times using Monte Carlo (MC) simulations. Next, the mean CFs were aggregated according to the exchanges' weighting defined by the ecoinvent activity. Finally, the relative 5th and 95th percentile unit CF deviations from the mean CF were calculated for every exchange and applied to the exchanges' average CF contributions to arrive at the activity's minimum (5th percentile) and maximum (95th percentile) refining emissions.

Building global battery-specific supply chains

Referring to the main method section "Constructing and mapping global battery supply chains", in constructing a global supply chain specific to batteries, we accounted for all mining products that could potentially be utilised in battery production. Specifically, all intermediate products that could be refined to battery chemicals were counted towards the global battery-specific supply chain and the y-axis of Figures 1c, f and h. This was only relevant for nickel, as the global nickel value chain involves many different end products of varying content grades, where the majority of mined nickel is used for steel production.[9]

The specific calculation for the nickel supply chain is found in "Country_Production_Shares_Mining.xlsx". Our analysis built on USGS,[10] BGS[11] and S&P Global data[1] and a 2020 McKinsey study.[9] Conceptually, it began, similar to lithium and cobalt, with 2022 USGS national production volumes, which were imputed from 2021 BGS data. We aggregated asset-level S&P Global data and combined them with USGS, BGS and DERA[12] information to determine the sulfide vs. laterite production shares of all relevant producing

countries. We noted from the McKinsey study that 46% of all mined nickel is – at some point – converted to Class 1 nickel, which we defined as an intermediate product that could be refined to produce nickel sulfate. Thus, combining this share with the laterite and sulfide shares of producing countries, the absolute sulfide and laterite production volumes for Class I nickel could be determined. We corroborated McKinsey's findings via a nickel industry expert interview and assumed that all mined nickel sulfide was converted to Class 1 nickel. Ultimately, we arrived at the global share of nickel laterite, which was converted to Class 1 nickel (33%).

Country- and deposit-specific data coverage shares in Figures 1c, f and h were computed by comparing the cumulative production of S&P Global assets with the battery-specific supply chain based on USGS and BGS data. In instances in which the cumulative production capacity of S&P Global assets surpassed the country- and deposit-specific volume in the previous step, the data coverage value was set to 100% of the country- and deposit-specific volume. This was the case for Australian and Zimbabwean spodumene production.

Turning emission curves into probability distributions

The emission curves shown in Figure 1 provided the basis for the construction of the emission probability density functions for each commodity. To this end, non-parametric Gaussian kernel estimates from the Python statistical library "scipy" were used, and the automatic bandwidth selection according to Scott's rule[13] was amended by the value .05, yielding a better balance between fit and smoothness. This approach was chosen in light of the highly varying shapes of the distributions. Supplementary Figure 2 below shows, in black, for each 4 + 1 (nickel including laterite) set of commodities, the assets' most likely CF (x-axis) and respective production volume (y-axis), normalised to 1. In green, the figure shows the assets' discrete CF distribution (N = 10,000 and normalised to 1), but considers the lower and upper margins of error using a triangular distribution and the most likely emission type as mode. Based on the green histograms, the above-described probability density functions (blue) were derived. Visible peaks in the functions correspond to (groups of) assets with large production volumes in Figures 1a, d and g.

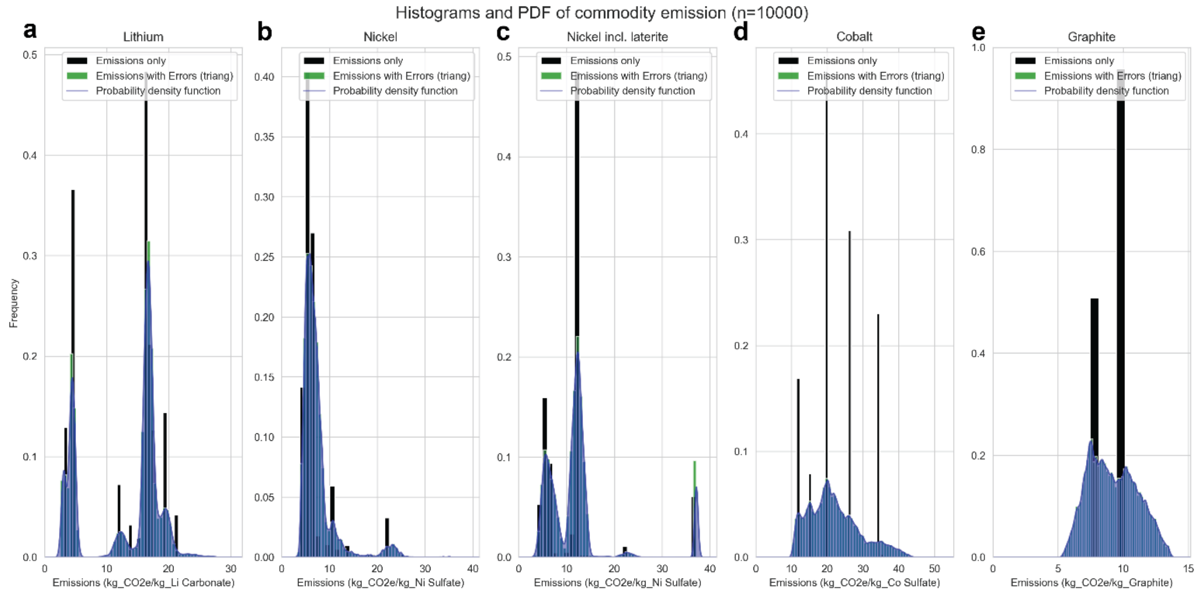

*Supplementary Figure 2: **Probability density functions based on emission curves.** Normalised most likely material CF values (black), discrete material CF distribution based on assets' most likely CF and margins of error (green) and continuous probability density functions (blue) for **a–e** lithium carbonate, nickel sulfate, nickel sulfate (incl. laterite), cobalt sulfate and graphite.*

LCA library Brightway2 and setting up Monte Carlo simulations

The refining step and margin of error calculations for battery materials, the entire battery CF Monte Carlo simulation, and all figures were generated using Python. The LCA library Brightway2 lies at the heart of the battery CF Monte Carlo simulation. ecoinvent 3.9.1 was loaded as a database into the Brightway2[14] project and supplemented with a standalone database where battery cell-specific activities were transferred from ecoinvent[15] into the foreground for streamlined access and modifications. While the repository used for this work has been made publicly accessible,[16] all files containing proprietary data (including S&P Global cost data, BNEF and ecoinvent) have been withheld for licensing reasons. For further information regarding the organisation of the repository and library versions, see the readme.md and the environment.yml files, respectively. Both can be found in the root directory of the repository.

Additivity of variances

We followed the analytical derivation available in the Supplementary Information of Hsiang et al.[17] for the additivity property and the corresponding variance interaction term. Let the carbon footprint be expressed as the function CF of a multitude of exchanges and variables. In this context, we can represent all exchanges that are not samples during the Monte Carlo simulation as a constant c. For simplicity, we show the additivity property for two variables Li, representing the unit CF of lithium carbonate, and Ni, representing the unit CF of nickel sulfate. The CF can consequently be written as supplementary equation (1):

$$\mathrm{CF} = \mathrm{f}(\mathrm{Li}, \mathrm{Ni}). (1)$$

Because Li and Ni are both independent random variables, the variance of the CF is supplementary equation (2):

$$\mathrm{Var}(\mathrm{CF}) = \mathrm{Var}(\mathrm{CF}|\mathrm{Li} = \mathrm{Li}_0) + \mathrm{Var}(\mathrm{CF}|\mathrm{Ni} = \mathrm{Ni}_0) + \Lambda\ (2)$$

where $\mathrm{Var}(\mathrm{CF}|\mathrm{Li} = \mathrm{Li}_0)$ refers to the variance of the CF function when the lithium variable is not varied (i.e., the variance driven through the nickel variable) and Λ to the interaction term. Solving for Λ yields supplementary equation (3):

$$\Lambda = \mathrm{Var}(\mathrm{CF}) - \mathrm{Var}(\mathrm{CF}|\mathrm{Li} = \mathrm{Li}_0) - \mathrm{Var}(\mathrm{CF}|\mathrm{Ni} = \mathrm{Ni}_0). (3)$$

As the impact calculation in LCA of multiple exchanges is additive, we can use the linear property and establish for CF = f(Li, Ni) that it is of the form CF = α + β*Li + γ*Ni. The same logic applies to the full Monte Carlo simulation, where the remaining variables cobalt, graphite and production location are also included. Calculating variances yields supplementary equations (4)–(6):

$$\mathrm{Var}(\mathrm{CF}) = \beta^2\mathrm{Var}(\mathrm{Li}) + \gamma^2\mathrm{Var}(\mathrm{Ni}), (4)$$

$$\mathrm{Var}(\mathrm{CF}|\mathrm{Li} = \mathrm{Li}_0) = \gamma^2\mathrm{Var}(\mathrm{Ni}), \text{and}\quad (5)$$

$$\mathrm{Var}(\mathrm{CF}|\mathrm{Ni} = \mathrm{Ni}_0) = \beta^2\mathrm{Var}(\mathrm{Li}).\quad (6)$$

Inserting supplementary equations (4)–(6) into supplementary equation (3) shows that the interaction term Λ is 0 for linear functions like CF. Thus, the variance of the total function can be expressed as the sum of the variances of individual variables.

List of expert interviews

Supplementary Table 2: List of expert interviews conducted in the context of this study

No.	Industry and position	Focus	Date	Duration
1	Raw materials industry, R&D manager	Technical	20.10.2022	45 min
2	Academia & raw materials industry, Materials sustainability manager	Technical & policy	18.01.2022	47 min
3	Raw materials industry, Public policy manager	Technical & policy	16.12.2022	70 min
4	Academia, Senior scientist	Technical & policy	05.10.2022	72 min
5	LCA consultancy, CEO	Technical & policy	05.10.2022	35 min
6	Strategy consultancy, Associate	Policy	19.12.2022	40 min
7	Academia, Research associate	Policy	16.12.2022	30 min
8	Strategy consultancy, Associate	Policy	16.12.2022	37 min
9	Chemical industry, Executive employee	Policy	14.12.2022	38 min
10	Academia, Research principal	Technical	12.09.2023	60 min
11	Chemical industry, Sustainability manager	Technical	25.09.2023	45 min
12	LCA consultancy, R&D manager	Technical & policy	25.10.2023	30 min
13	Data provider, Senior analyst	Technical	31.10.2023	45 min
14	Battery industry, LCA analyst	Technical & policy	03.11.2023	50 min
15	Battery consultancy, CEO	Technical	03.11.2023	60 min
16	Battery industry association, Manager	Policy	11.12.2023	45 min
17	Cathode producer, CEO	Technical	29.01.2024	10 min

Supplementary Table 3: Statistical values (5th, 50th, 95th percentiles, standard deviation and variance) for datasets underlying Figures 2 and 3 and Supplementary Figures 7,8, and 9. Region acronyms: CN: China, US: United States, EU: Europe, KRJP: Korea + Japan.

	Region	5th percentile [kg_{CO2} kWh^{-1}]	50th percentile [kg_{CO2} kWh^{-1}]	95th percentile [kg_{CO2} kWh^{-1}]	Standard deviation [kg_{CO2} kWh^{-1}]	Variance [$(kg_{CO2}\ kWh^{-1})^2$]
NMC	all	54.90	68.13	84.95	9.88	97.57
	CN	58.64	70.90	86.50	9.46	89.49
	US	54.37	64.89	77.85	8.41	70.65
	EU	51.43	63.19	80.48	9.48	89.84
	KRJP	59.23	68.34	82.15	8.42	70.94
LFP	all	53.83	62.31	68.60	4.59	21.05
	CN	54.16	62.48	68.69	4.54	20.65
	US	51.63	59.44	64.51	4.05	16.38
	EU	50.28	58.43	64.73	4.47	19.95
	KRJP	53.93	61.48	66.57	3.98	15.82
NMC (laterite included)	all	58.52	74.39	114.52	14.75	217.45
	CN	62.14	77.18	117.99	14.39	207.14
	US	57.79	70.88	110.45	13.34	178.05
	EU	54.92	69.10	111.09	14.76	217.73
	KRJP	61.69	75.12	115.48	13.51	182.42
LFP (solid state)	all	53.51	61.09	67.12	4.25	18.10
	CN	53.92	61.24	67.19	4.19	17.59
	US	51.33	58.21	63.09	3.61	13.05
	EU	49.75	57.21	63.56	4.16	17.32
	KRJP	53.44	60.24	64.96	3.54	12.55
LFP (hydrothermal)	all	56.43	72.94	80.37	8.12	65.88
	CN	56.61	73.12	80.41	8.09	65.39
	US	54.51	70.62	77.20	7.84	61.39
	EU	53.17	69.59	77.50	8.06	64.91
	KRJP	56.36	72.43	78.85	7.82	61.19

Supplementary Note 1 Breakdown of CF contributions

For the sake of brevity, these supplementary tables contain only the CF breakdowns supporting the main study Figures 2c and 3c. The breakdown of the nickel-based cathodes (NMC) scenario using the nickel emission curve, excluding laterite deposits and iron-based cathodes (LFP) batteries with purely solid-state and hydrothermal active material synthesis, can be found in the accompanying Excel file “CF_breakdown (Fig2c_3c)”. Note that for the main figures, activities (rows in the tables below) were grouped according to minimum thresholds and other criteria defined in “dict_labels_for_barcharts.xlsx” located in the repository accompanying this paper.

Supplementary Table 4: Disaggregated relative and absolute CF contributions of exchanges and activities for the production of NMC811 battery cells (including laterite).

Exchange	Abs. CF contribution (weighted) [kg$_{CO2}$ kWh^{-1}]	Rel. CF contribution (weighted) [%]	Exchange hierarchy
NMC_battery_cell production	74.39	100.00	$NMC_battery_cell production
Graphite production	7.26	9.75	$NMC_battery_cell production$Anode_paste_for_NMC production$Graphite production
market for carbon black	0.02	0.03	$NMC_battery_cell production$Anode_paste_for_NMC production$market for carbon black
market for carboxymethyl cellulose, powder	0.11	0.14	$NMC_battery_cell production$Anode_paste_for_NMC production$market for carboxymethyl cellulose, powder
market for chemical factory, organics	0.05	0.07	$NMC_battery_cell production$Anode_paste_for_NMC production$market for chemical factory, organics
market for electricity, medium voltage	0.26	0.35	$NMC_battery_cell production$Anode_paste_for_NMC production$market for electricity, medium voltage
market for heat, district or industrial, natural gas	0.28	0.38	$NMC_battery_cell production$Anode_paste_for_NMC production$market for heat, district or industrial, natural gas
market for latex	0.05	0.06	$NMC_battery_cell production$Anode_paste_for_NMC production$market for latex
market for silicon, metallurgical grade	0.19	0.26	$NMC_battery_cell production$Anode_paste_for_NMC production$market for silicon, metallurgical grade
market for wastewater, average	0.00	0.00	$NMC_battery_cell production$Anode_paste_for_NMC production$market for wastewater, average
market for water, deionised	0.00	0.00	$NMC_battery_cell production$Anode_paste_for_NMC production$market for water, deionised
Calcium II	0.00	0.00	$NMC_battery_cell production$NMC_cathode_paste production$Li_NMC_active_material production$Li_hydroxide production$Calcium II
Carbonate	0.00	0.00	$NMC_battery_cell production$NMC_cathode_paste production$Li_NMC_active_material production$Li_hydroxide production$Carbonate
Hydroxide	0.00	0.00	$NMC_battery_cell production$NMC_cathode_paste production$Li_NMC_active_material production$Li_hydroxide production$Hydroxide
Lithium I	0.00	0.00	$NMC_battery_cell production$NMC_cathode_paste production$Li_NMC_active_material production$Li_hydroxide production$Lithium I

Lithium_carbonate production	7.65	10.29	$NMC_battery_cell production$NMC_cathode_paste production$Li_NMC_active_material production$Li_hydroxide production$Lithium_carbonate production
Water	0.00	0.00	$NMC_battery_cell production$NMC_cathode_paste production$Li_NMC_active_material production$Li_hydroxide production$Water
Water	0.00	0.00	$NMC_battery_cell production$NMC_cathode_paste production$Li_NMC_active_material production$Li_hydroxide production$Water
Water, cooling, unspecified natural origin	0.00	0.00	$NMC_battery_cell production$NMC_cathode_paste production$Li_NMC_active_material production$Li_hydroxide production$Water, cooling, unspecified natural origin
Water, river	0.00	0.00	$NMC_battery_cell production$NMC_cathode_paste production$Li_NMC_active_material production$Li_hydroxide production$Water, river
Water, well, in ground	0.00	0.00	$NMC_battery_cell production$NMC_cathode_paste production$Li_NMC_active_material production$Li_hydroxide production$Water, well, in ground
market for chemical factory, organics	0.03	0.05	$NMC_battery_cell production$NMC_cathode_paste production$Li_NMC_active_material production$Li_hydroxide production$market for chemical factory, organics
market for electricity, medium voltage	0.15	0.20	$NMC_battery_cell production$NMC_cathode_paste production$Li_NMC_active_material production$Li_hydroxide production$market for electricity, medium voltage
market for heat, from steam, in chemical industry	0.00	0.00	$NMC_battery_cell production$NMC_cathode_paste production$Li_NMC_active_material production$Li_hydroxide production$market for heat, from steam, in chemical industry
market for heat, from steam, in chemical industry	0.01	0.02	$NMC_battery_cell production$NMC_cathode_paste production$Li_NMC_active_material production$Li_hydroxide production$market for heat, from steam, in chemical industry
market for inert waste	0.00	0.01	$NMC_battery_cell production$NMC_cathode_paste production$Li_NMC_active_material production$Li_hydroxide production$market for inert waste
market for inert waste	0.00	0.00	$NMC_battery_cell production$NMC_cathode_paste production$Li_NMC_active_material production$Li_hydroxide production$market for inert waste
market for inert waste	0.00	0.00	$NMC_battery_cell production$NMC_cathode_paste production$Li_NMC_active_material production$Li_hydroxide production$market for inert waste
market for lime, hydrated, loose weight	0.00	0.01	$NMC_battery_cell production$NMC_cathode_paste production$Li_NMC_active_material production$Li_hydroxide production$market for lime, hydrated, loose weight
market for lime, hydrated, loose weight	0.54	0.72	$NMC_battery_cell production$NMC_cathode_paste production$Li_NMC_active_material production$Li_hydroxide production$market for lime, hydrated, loose weight
market for wastewater, average	0.00	0.00	$NMC_battery_cell production$NMC_cathode_paste production$Li_NMC_active_material production$Li_hydroxide production$market for wastewater, average
market for wastewater, average	0.00	0.00	$NMC_battery_cell production$NMC_cathode_paste production$Li_NMC_active_material production$Li_hydroxide production$market for wastewater, average
market for wastewater, average	0.00	0.00	$NMC_battery_cell production$NMC_cathode_paste production$Li_NMC_active_material production$Li_hydroxide production$market for wastewater, average
market for wastewater, average	0.00	0.00	$NMC_battery_cell production$NMC_cathode_paste production$Li_NMC_active_material production$Li_hydroxide production$market for wastewater, average
market group for heat, district or industrial, natural gas	0.05	0.07	$NMC_battery_cell production$NMC_cathode_paste production$Li_NMC_active_material production$Li_hydroxide production$market group for heat, district or industrial, natural gas
market group for tap water	0.00	0.00	$NMC_battery_cell production$NMC_cathode_paste production$Li_NMC_active_material production$Li_hydroxide production$market group for tap water
Ammonia	0.00	0.00	$NMC_battery_cell production$NMC_cathode_paste production$Li_NMC_active_material production$NMC_hydroxide production$Ammonia
Co_sulfate production	4.99	6.71	$NMC_battery_cell production$NMC_cathode_paste production$Li_NMC_active_material production$NMC_hydroxide production$Co_sulfate production

Ni_sulfate production	19.33	25.98	$NMC_battery_cell production$NMC_cathode_paste production$Li_NMC_active_material production$NMC_hydroxide production$Ni_sulfate production
Water, cooling, unspecified natural origin	0.00	0.00	$NMC_battery_cell production$NMC_cathode_paste production$Li_NMC_active_material production$NMC_hydroxide production$Water, cooling, unspecified natural origin
market for ammonia, anhydrous, liquid	0.04	0.05	$NMC_battery_cell production$NMC_cathode_paste production$Li_NMC_active_material production$NMC_hydroxide production$market for ammonia, anhydrous, liquid
market for chemical factory, organics	0.15	0.20	$NMC_battery_cell production$NMC_cathode_paste production$Li_NMC_active_material production$NMC_hydroxide production$market for chemical factory, organics
market for heat, district or industrial, natural gas	2.03	2.72	$NMC_battery_cell production$NMC_cathode_paste production$Li_NMC_active_material production$NMC_hydroxide production$market for heat, district or industrial, natural gas
market for manganese sulfate	0.19	0.25	$NMC_battery_cell production$NMC_cathode_paste production$Li_NMC_active_material production$NMC_hydroxide production$market for manganese sulfate
market for sodium hydroxide, without water, in 50% solution state	1.50	2.02	$NMC_battery_cell production$NMC_cathode_paste production$Li_NMC_active_material production$NMC_hydroxide production$market for sodium hydroxide, without water, in 50% solution state
market for wastewater, unpolluted	0.00	0.00	$NMC_battery_cell production$NMC_cathode_paste production$Li_NMC_active_material production$NMC_hydroxide production$market for wastewater, unpolluted
Water	0.00	0.00	$NMC_battery_cell production$NMC_cathode_paste production$Li_NMC_active_material production$Water
market for chemical factory, organics	0.16	0.22	$NMC_battery_cell production$NMC_cathode_paste production$Li_NMC_active_material production$market for chemical factory, organics
market for electricity, medium voltage	6.56	8.82	$NMC_battery_cell production$NMC_cathode_paste production$Li_NMC_active_material production$market for electricity, medium voltage
N-methyl-2-pyrrolidone	0.00	0.00	$NMC_battery_cell production$NMC_cathode_paste production$N-methyl-2-pyrrolidone
market for N-methyl-2-pyrrolidone	0.06	0.08	$NMC_battery_cell production$NMC_cathode_paste production$market for N-methyl-2-pyrrolidone
market for carbon black	0.09	0.12	$NMC_battery_cell production$NMC_cathode_paste production$market for carbon black
market for chemical factory, organics	0.09	0.12	$NMC_battery_cell production$NMC_cathode_paste production$market for chemical factory, organics
market for electricity, medium voltage	0.45	0.60	$NMC_battery_cell production$NMC_cathode_paste production$market for electricity, medium voltage
market for heat, district or industrial, natural gas	1.61	2.17	$NMC_battery_cell production$NMC_cathode_paste production$market for heat, district or industrial, natural gas
market for polyvinylfluoride	0.60	0.81	$NMC_battery_cell production$NMC_cathode_paste production$market for polyvinylfluoride
market for aluminium collector foil, for Li-ion battery	1.79	2.40	$NMC_battery_cell production$market for aluminium collector foil, for Li-ion battery
market for aluminium, wrought alloy	1.57	2.11	$NMC_battery_cell production$market for aluminium, wrought alloy
market for battery separator	2.51	3.37	$NMC_battery_cell production$market for battery separator
market for chemical factory, organics	0.24	0.32	$NMC_battery_cell production$market for chemical factory, organics
market for copper collector foil, for Li-ion battery	4.33	5.82	$NMC_battery_cell production$market for copper collector foil, for Li-ion battery
market for copper, anode	0.80	1.08	$NMC_battery_cell production$market for copper, anode

market for electricity, medium voltage	3.18	4.28	$NMC_battery_cell production$market for electricity, medium voltage
market for electrolyte, for Li-ion battery	3.24	4.35	$NMC_battery_cell production$market for electrolyte, for Li-ion battery
market for extrusion, plastic film	0.01	0.01	$NMC_battery_cell production$market for extrusion, plastic film
market for heat, district or industrial, natural gas	2.02	2.72	$NMC_battery_cell production$market for heat, district or industrial, natural gas
market for polyethylene terephthalate, granulate, amorphous	0.04	0.05	$NMC_battery_cell production$market for polyethylene terephthalate, granulate, amorphous
market for polypropylene, granulate	0.01	0.01	$NMC_battery_cell production$market for polypropylene, granulate
market for sheet rolling, aluminium	0.07	0.10	$NMC_battery_cell production$market for sheet rolling, aluminium
market for sheet rolling, copper	0.07	0.10	$NMC_battery_cell production$market for sheet rolling, copper
market for sheet rolling, aluminium	0.11	0.17	$LFP_battery_cell production$market for sheet rolling, aluminium
market for sheet rolling, copper	0.10	0.16	$LFP_battery_cell production$market for sheet rolling, copper

Supplementary Table 5: Disaggregated relative and absolute CF contributions of exchanges and activities for the production of LFP battery cells (90% solid-state share).

**Exchange**	**Abs. CF contribution (weighted) [kg$_{CO2}$ kWh^{-1}]**	**Rel. CF contribution (weighted) [%]**	**Exchange hierarchy**
LFP_battery_cell production	62.31	100.00	$LFP_battery_cell production
Graphite production	9.16	14.70	$LFP_battery_cell production$Anode_paste_for_LFP production$Graphite production
market for carbon black	0.03	0.04	$LFP_battery_cell production$Anode_paste_for_LFP production$market for carbon black
market for carboxymethyl cellulose, powder	0.13	0.22	$LFP_battery_cell production$Anode_paste_for_LFP production$market for carboxymethyl cellulose, powder
market for chemical factory, organics	0.07	0.11	$LFP_battery_cell production$Anode_paste_for_LFP production$market for chemical factory, organics
market for electricity, medium voltage	0.39	0.62	$LFP_battery_cell production$Anode_paste_for_LFP production$market for electricity, medium voltage
market for heat, district or industrial, natural gas	0.33	0.53	$LFP_battery_cell production$Anode_paste_for_LFP production$market for heat, district or industrial, natural gas
market for latex	0.06	0.10	$LFP_battery_cell production$Anode_paste_for_LFP production$market for latex
market for wastewater, average	0.00	0.00	$LFP_battery_cell production$Anode_paste_for_LFP production$market for wastewater, average
market for water, deionised	0.00	0.00	$LFP_battery_cell production$Anode_paste_for_LFP production$market for water, deionised

Calcium II	0.00	0.00	$LFP_battery_cell production$LFP_cathode_paste production$Market_LFP production$LFP_hydrothermal production$Li_hydroxide production$Calcium II
Carbonate	0.00	0.00	$LFP_battery_cell production$LFP_cathode_paste production$Market_LFP production$LFP_hydrothermal production$Li_hydroxide production$Carbonate
Hydroxide	0.00	0.00	$LFP_battery_cell production$LFP_cathode_paste production$Market_LFP production$LFP_hydrothermal production$Li_hydroxide production$Hydroxide
Lithium I	0.00	0.00	$LFP_battery_cell production$LFP_cathode_paste production$Market_LFP production$LFP_hydrothermal production$Li_hydroxide production$Lithium I
Lithium_carbonate production	1.83	2.94	$LFP_battery_cell production$LFP_cathode_paste production$Market_LFP production$LFP_hydrothermal production$Li_hydroxide production$Lithium_carbonate production
Water	0.00	0.00	$LFP_battery_cell production$LFP_cathode_paste production$Market_LFP production$LFP_hydrothermal production$Li_hydroxide production$Water
Water	0.00	0.00	$LFP_battery_cell production$LFP_cathode_paste production$Market_LFP production$LFP_hydrothermal production$Li_hydroxide production$Water
Water, cooling, unspecified natural origin	0.00	0.00	$LFP_battery_cell production$LFP_cathode_paste production$Market_LFP production$LFP_hydrothermal production$Li_hydroxide production$Water, cooling, unspecified natural origin
Water, river	0.00	0.00	$LFP_battery_cell production$LFP_cathode_paste production$Market_LFP production$LFP_hydrothermal production$Li_hydroxide production$Water, river
Water, well, in ground	0.00	0.00	$LFP_battery_cell production$LFP_cathode_paste production$Market_LFP production$LFP_hydrothermal production$Li_hydroxide production$Water, well, in ground
market for chemical factory, organics	0.01	0.01	$LFP_battery_cell production$LFP_cathode_paste production$Market_LFP production$LFP_hydrothermal production$Li_hydroxide production$market for chemical factory, organics
market for electricity, medium voltage	0.04	0.06	$LFP_battery_cell production$LFP_cathode_paste production$Market_LFP production$LFP_hydrothermal production$Li_hydroxide production$market for electricity, medium voltage
market for heat, from steam, in chemical industry	0.00	0.00	$LFP_battery_cell production$LFP_cathode_paste production$Market_LFP production$LFP_hydrothermal production$Li_hydroxide production$market for heat, from steam, in chemical industry
market for heat, from steam, in chemical industry	0.00	0.00	$LFP_battery_cell production$LFP_cathode_paste production$Market_LFP production$LFP_hydrothermal production$Li_hydroxide production$market for heat, from steam, in chemical industry
market for inert waste	0.00	0.00	$LFP_battery_cell production$LFP_cathode_paste production$Market_LFP production$LFP_hydrothermal production$Li_hydroxide production$market for inert waste
market for inert waste	0.00	0.00	$LFP_battery_cell production$LFP_cathode_paste production$Market_LFP production$LFP_hydrothermal production$Li_hydroxide production$market for inert waste
market for inert waste	0.00	0.00	$LFP_battery_cell production$LFP_cathode_paste production$Market_LFP production$LFP_hydrothermal production$Li_hydroxide production$market for inert waste
market for lime, hydrated, loose weight	0.00	0.00	$LFP_battery_cell production$LFP_cathode_paste production$Market_LFP production$LFP_hydrothermal production$Li_hydroxide production$market for lime, hydrated, loose weight
market for lime, hydrated, loose weight	0.12	0.18	$LFP_battery_cell production$LFP_cathode_paste production$Market_LFP production$LFP_hydrothermal production$Li_hydroxide production$market for lime, hydrated, loose weight
market for wastewater, average	0.00	0.00	$LFP_battery_cell production$LFP_cathode_paste production$Market_LFP production$LFP_hydrothermal production$Li_hydroxide production$market for wastewater, average
market for wastewater, average	0.00	0.00	$LFP_battery_cell production$LFP_cathode_paste production$Market_LFP production$LFP_hydrothermal production$Li_hydroxide production$market for wastewater, average
market for wastewater, average	0.00	0.00	$LFP_battery_cell production$LFP_cathode_paste production$Market_LFP production$LFP_hydrothermal production$Li_hydroxide production$market for wastewater, average
market for wastewater, average	0.00	0.00	$LFP_battery_cell production$LFP_cathode_paste production$Market_LFP production$LFP_hydrothermal production$Li_hydroxide production$market for wastewater, average

market group for heat, district or industrial, natural gas	0.01	0.02	$LFP_battery_cell production$LFP_cathode_paste production$Market_LFP production$LFP_hydrothermal production$Li_hydroxide production$market group for heat, district or industrial, natural gas
market group for tap water	0.00	0.00	$LFP_battery_cell production$LFP_cathode_paste production$Market_LFP production$LFP_hydrothermal production$Li_hydroxide production$market group for tap water
Water	0.00	0.00	$LFP_battery_cell production$LFP_cathode_paste production$Market_LFP production$LFP_hydrothermal production$Water
market for chemical factory, organics	0.02	0.03	$LFP_battery_cell production$LFP_cathode_paste production$Market_LFP production$LFP_hydrothermal production$market for chemical factory, organics
market for heat, district or industrial, natural gas	0.21	0.34	$LFP_battery_cell production$LFP_cathode_paste production$Market_LFP production$LFP_hydrothermal production$market for heat, district or industrial, natural gas
market for iron sulfate	0.05	0.07	$LFP_battery_cell production$LFP_cathode_paste production$Market_LFP production$LFP_hydrothermal production$market for iron sulfate
market for phosphoric acid, industrial grade, without water, in 85% solution state	0.14	0.23	$LFP_battery_cell production$LFP_cathode_paste production$Market_LFP production$LFP_hydrothermal production$market for phosphoric acid, industrial grade, without water, in 85% solution state
market for tap water	0.01	0.01	$LFP_battery_cell production$LFP_cathode_paste production$Market_LFP production$LFP_hydrothermal production$market for tap water
market for wastewater, average	0.00	0.00	$LFP_battery_cell production$LFP_cathode_paste production$Market_LFP production$LFP_hydrothermal production$market for wastewater, average
Ammonia	0.00	0.00	$LFP_battery_cell production$LFP_cathode_paste production$Market_LFP production$LFP_solid_state production$Ammonia
Carbon dioxide, fossil	0.26	0.41	$LFP_battery_cell production$LFP_cathode_paste production$Market_LFP production$LFP_solid_state production$Carbon dioxide, fossil
Lithium_carbonate production	6.42	10.30	$LFP_battery_cell production$LFP_cathode_paste production$Market_LFP production$LFP_solid_state production$Lithium_carbonate production
Oxygen	0.00	0.00	$LFP_battery_cell production$LFP_cathode_paste production$Market_LFP production$LFP_solid_state production$Oxygen
Water	0.00	0.00	$LFP_battery_cell production$LFP_cathode_paste production$Market_LFP production$LFP_solid_state production$Water
market for chemical factory, organics	0.21	0.33	$LFP_battery_cell production$LFP_cathode_paste production$Market_LFP production$LFP_solid_state production$market for chemical factory, organics
market for diammonium phosphate	3.22	5.17	$LFP_battery_cell production$LFP_cathode_paste production$Market_LFP production$LFP_solid_state production$market for diammonium phosphate
market for electricity, medium voltage	1.08	1.73	$LFP_battery_cell production$LFP_cathode_paste production$Market_LFP production$LFP_solid_state production$market for electricity, medium voltage
market for magnetite	0.83	1.33	$LFP_battery_cell production$LFP_cathode_paste production$Market_LFP production$LFP_solid_state production$market for magnetite
N-methyl-2-pyrrolidone	0.00	0.00	$LFP_battery_cell production$LFP_cathode_paste production$N-methyl-2-pyrrolidone
market for N-methyl-2-pyrrolidone	0.15	0.25	$LFP_battery_cell production$LFP_cathode_paste production$market for N-methyl-2-pyrrolidone
market for carbon black	0.33	0.52	$LFP_battery_cell production$LFP_cathode_paste production$market for carbon black
market for chemical factory, organics	0.14	0.22	$LFP_battery_cell production$LFP_cathode_paste production$market for chemical factory, organics
market for electricity, medium voltage	0.64	1.03	$LFP_battery_cell production$LFP_cathode_paste production$market for electricity, medium voltage
market for heat, district or industrial, natural gas	2.58	4.14	$LFP_battery_cell production$LFP_cathode_paste production$market for heat, district or industrial, natural gas

market for polyvinylfluoride	1.83	2.94	$LFP_battery_cell production$LFP_cathode_paste production$market for polyvinylfluoride
market for aluminium collector foil, for Li-ion battery	4.87	7.81	$LFP_battery_cell production$market for aluminium collector foil, for Li-ion battery
market for aluminium, wrought alloy	2.25	3.61	$LFP_battery_cell production$market for aluminium, wrought alloy
market for battery separator	3.36	5.40	$LFP_battery_cell production$market for battery separator
market for chemical factory, organics	0.38	0.60	$LFP_battery_cell production$market for chemical factory, organics
market for copper collector foil, for Li-ion battery	5.98	9.60	$LFP_battery_cell production$market for copper collector foil, for Li-ion battery
market for copper, anode	1.11	1.78	$LFP_battery_cell production$market for copper, anode
market for electricity, medium voltage	4.72	7.58	$LFP_battery_cell production$market for electricity, medium voltage
market for electrolyte, for Li-ion battery	6.66	10.69	$LFP_battery_cell production$market for electrolyte, for Li-ion battery
market for extrusion, plastic film	0.01	0.02	$LFP_battery_cell production$market for extrusion, plastic film
market for heat, district or industrial, natural gas	2.40	3.86	$LFP_battery_cell production$market for heat, district or industrial, natural gas
market for polyethylene terephthalate, granulate, amorphous	0.06	0.10	$LFP_battery_cell production$market for polyethylene terephthalate, granulate, amorphous
market for polypropylene, granulate	0.02	0.03	$LFP_battery_cell production$market for polypropylene, granulate
market for sheet rolling, aluminium	0.11	0.17	$LFP_battery_cell production$market for sheet rolling, aluminium
market for sheet rolling, copper	0.10	0.16	$LFP_battery_cell production$market for sheet rolling, copper

Supplementary Table 6: Tabulated variance breakdown for all chemistries and variations. Variance of MC simulations with single-parameter sampling and sums thereof. The sum is defined as 100% (indicated by ≡) and contrasted to the variance of the MC simulation with simultaneous parameter sampling.

		Production location (Electricity)	Nickel distribution	Lithium distribution	Cobalt distribution	Graphite distribution	TOTAL (individual MC runs)	TOTAL (all parameters sampled)
NMC	Variance (abs.) [(kg_{CO2} kWh^{-1})^2]	26.99	50.61	12.39	3.13	2.31	95.42	97.57
	Variance (rel.) [%]	28.28	53.04	12.98	3.28	2.42	≡ 100.00	102.25
LFP	Variance (abs.) [(kg_{CO2} kWh^{-1})^2]	5.15	na	11.65	na	3.97	20.77	21.05
	Variance (rel.) [%]	24.78	na	56.11	na	19.11	≡ 100.00	101.37
NMC (laterite include	Variance (abs.) [(kg_{CO2} kWh^{-1})^2]	27.71	164.90	12.37	3.23	2.31	210.52	217.45
	Variance (rel.) [%]	13.16	78.33	5.88	1.53	1.10	≡ 100.00	103.29
LFP (solid state)	Variance (abs.) [(kg_{CO2} kWh^{-1})^2]	5.42	na	8.63	na	3.92	17.98	18.10
	Variance (rel.) [%]	30.18	na	48.03	na	21.79	≡ 100.00	100.68
LFP (hydrother mal)	Variance (abs.) [(kg_{CO2} kWh^{-1})^2]	4.12	na	57.82	na	3.92	65.85	65.88
	Variance (rel.) [%]	6.25	na	87.80	na	5.95	≡ 100.00	100.05

Supplementary Note 2 Additional emission curves

While Panels a and b of Supplementary Figure 3 show that the CF of synthetic graphite production ranges from around 6 to 14 kg_{CO2} $kg_{Graphite}^{-1}$, expert interviews revealed that the CF can reach up to 25 kg_{CO2} $kg_{Graphite}^{-1}$ in extreme cases. However, we did not incorporate estimates obtained from expert interviews into this work's CF database (Supplementary Note 4) to maintain methodological consistency and ensure reproducibility. Nonetheless, this strong discrepancy is testament to the highly insufficient data coverage and calls for more comprehensive research on battery-grade graphite.

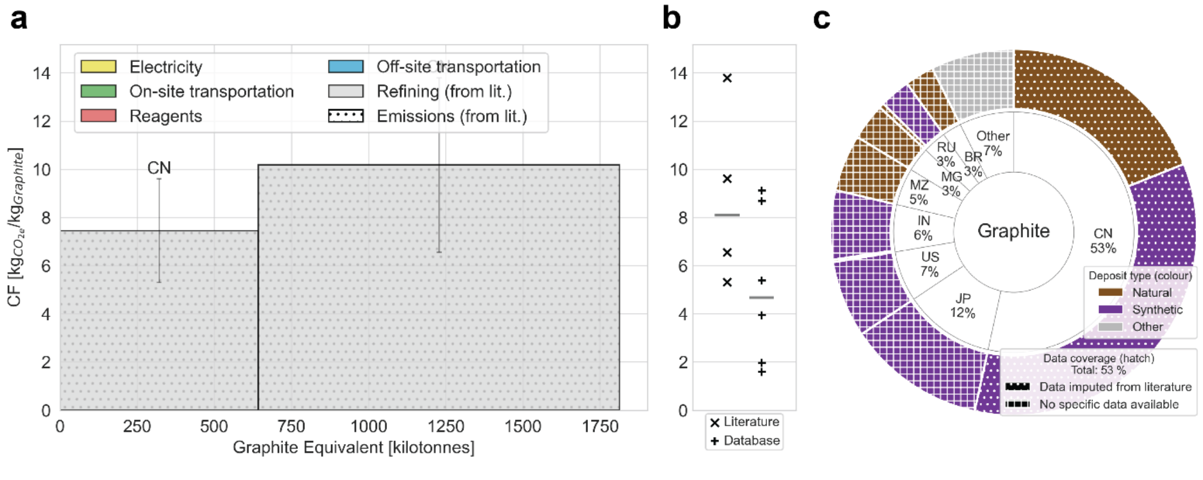

*Supplementary Figure 3: **Emission curves, literature and database carbon footprint values and 2022 supply chain for graphite. a** Emission curves for graphite. Dotted grey bar segments refer to imputation-based data sources. Production volumes on the x-axis are indicated in chemical equivalents, i.e. encompassing all intermediate products that could be refined to battery-grade chemicals. Whiskers indicate the minimum and maximum values. **b** Literature and database (ecoinvent 3.9.1 and GREET 2021) values. Horizontal lines indicate respective medians.*

See Supplementary Note 4 for the underlying review work. ***c*** *Doughnut charts of global 2022 battery-specific supply curves, i.e. encompassing all intermediate products that could be refined to battery-grade chemicals. The inner ring refers to country mining shares, while the outer ring indicates deposit types and data coverage. Dotted bar segments refer to imputed data, and chequered segments indicate no data availability.* ***Country acronyms*** *BR: Brazil, CN: China, IN: India, JP: Japan, MG: Madagascar, MZ: Mozambique, RU: Russia, US: United States of America. Source data are provided as a Source Data file.*

Supplementary Figure 4 details nickel sulfate CF emission curves with excluded laterite deposits. In panel a, colored segments represent model data, while grey areas denote literature-based data. Panel b similarly compares CF values from literature, databases, and reports, with medians marked. Panel c provides a breakdown of nickel supply for batteries, showing Indonesia as the largest supplier of nickel laterites (whose data is not covered vis-à-vis Figure 1d, e and f in the main manuscript), with data gaps marked in chequered segments.

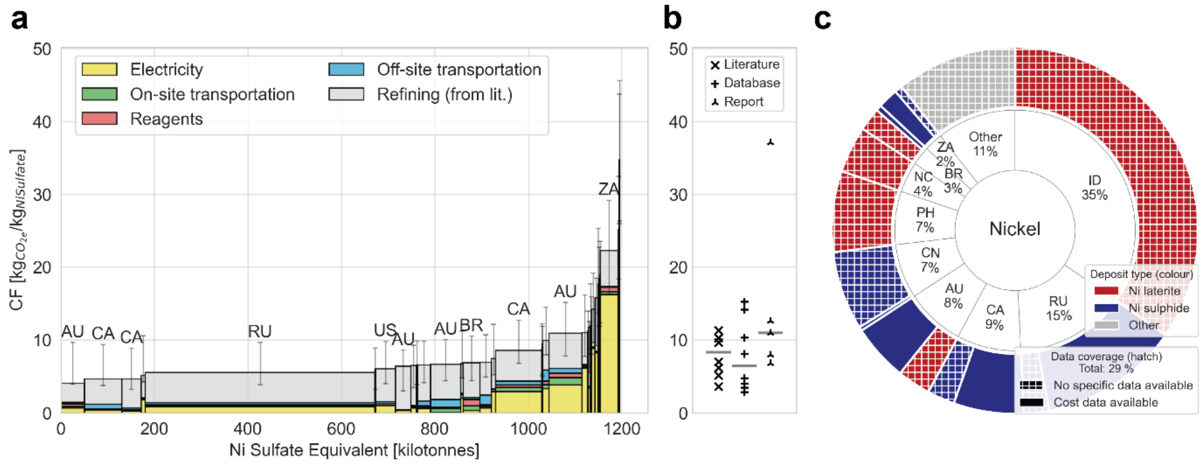

Supplementary Figure 4: ***Emission curves, literature and database carbon footprint values and 2022 supply chain for nickel sulfate equivalent, excluding laterite ore deposits. a*** *Emission curves for nickel sulfate, excluding laterite mining. Coloured bar segments correspond to modelled data sources and solid grey to literature-based data sources. Production volumes on the x-axis are indicated in chemical equivalents, i.e. encompassing all intermediate products that could be refined to battery-grade chemicals. All sulfate products are in their anhydrous form. Whiskers indicate the minimum and maximum values.* ***b*** *Literature, report and database (ecoinvent 3.9.1 and GREET 2021) values. Horizontal lines indicate respective medians. See Supplementary Note 4 for the underlying review work.* ***c*** *Doughnut charts of global 2022 battery-specific supply curves, i.e. encompassing all intermediate products that could be refined to battery-grade chemicals. The inner ring refers to country mining shares, while the outer ring indicates deposit types and data coverage. Chequered bar segments indicate no data availability. Country acronyms: AU: Australia, BR: Brazil, CA: Canada, CN: China, ID: Indonesia, NC: New Caledonia (sui generis collectivity of France), PH: Philippines, RU: Russia, ZA: South Africa. Source data are provided as a Source Data file.*

Supplementary Figures 5 and 6 display CF probability distributions in kg-units for NMC811 and LFP battery cells, respectively, produced across different global regions. Both figures show China's dominating share of production, with China accounting for 59.5% of NMC811 cells and 95.2% of LFP cells, impacting the CF distribution across regions due to regional emission intensities. These two figures correspond to Figures 2 and 3 of the main manuscript by converting the kWh-basis to a kg-basis using the respective cell energy densities.

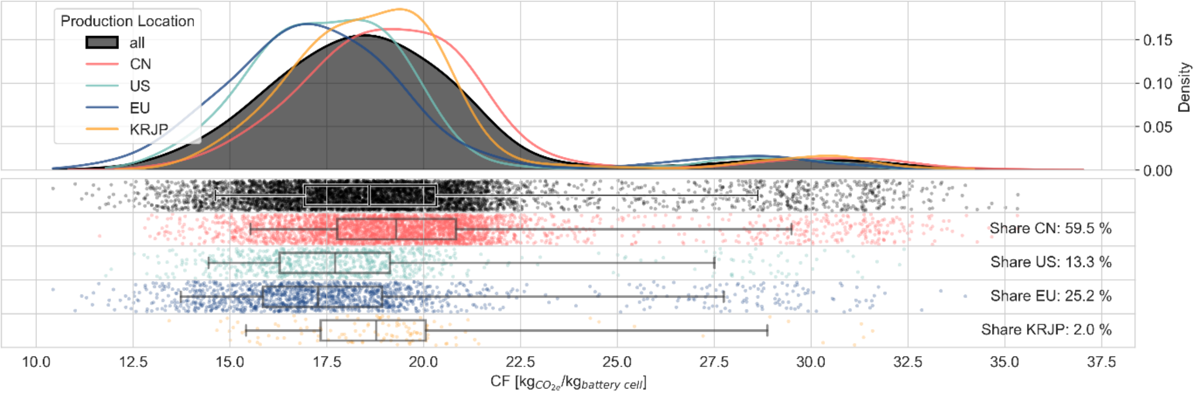

*Supplementary Figure 5: **Probability density distribution and jitter plot for LIB cells with NMC811 cathodes, including laterite ore deposits in per-kg units.** Caption comments from Figure 2 apply. Source data are provided as a Source Data file.*

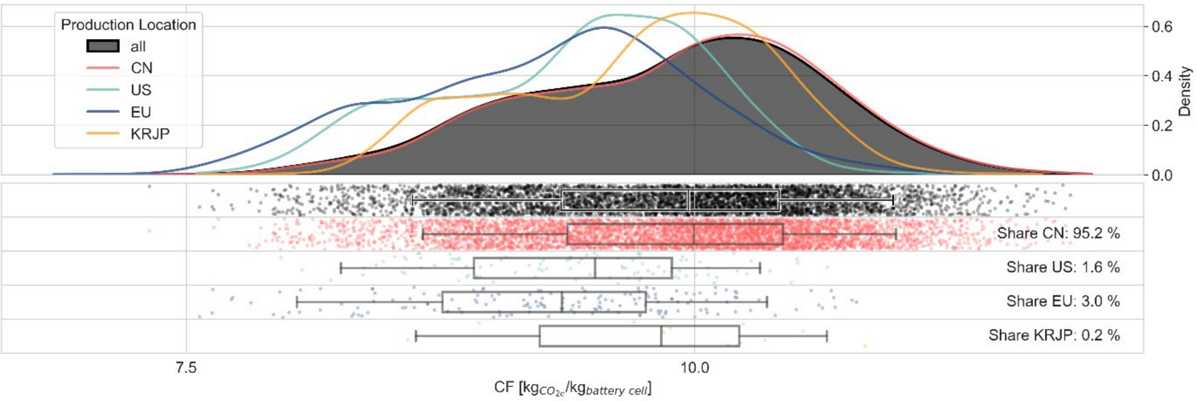

*Supplementary Figure 6: **Probability density distribution and jitter plot for LIB cells with LFP cathodes in per kg units.** Caption comments from Figure 3 apply. Source data are provided as a Source Data file.*

Supplementary Note 3 Additional CF distributions

In Supplementary Figures 7-9, we see the CF distributions and contributions for LIB cells, focusing on specific cathode types and synthesis methods.

Supplementary Figure 7 illustrates the CF profile for LIB cells with NMC811 cathodes, excluding laterite ore deposits. As laterite are excluded, in panel a, probability density functions do not feature the second mode at around 120 kg_{CO2e} kWh^{-1} visible in Figure 2 in the main manuscript. Panel b highlights major CF drivers, including nickel sulfate (18.0%), lithium carbonate (12.2%), and graphite (10.8%). The decreased contribution of nickel sulfate compared to Figure 2 in the main manuscript is attributable to the exclusion of laterite ore. Nonetheless, panel c shows that nickel sulfate is still the largest contributor to CF variance, emphasizing the material's critical role in CF outcomes.

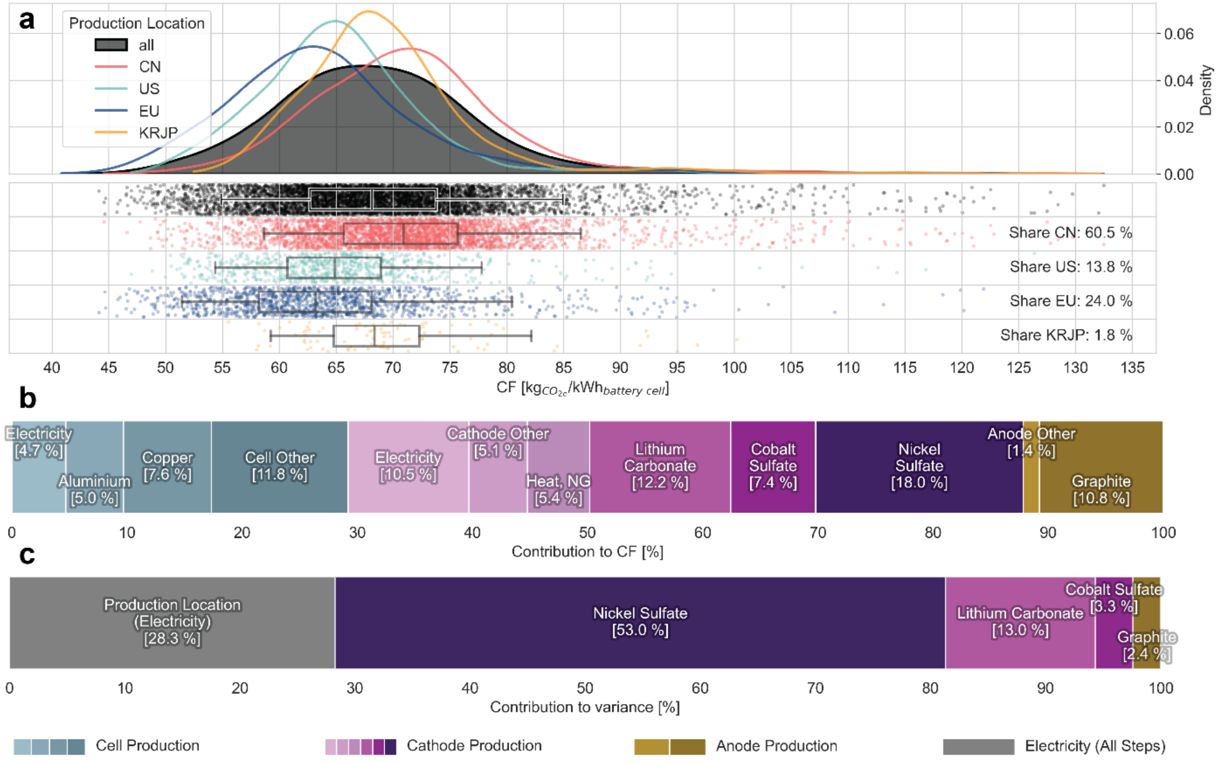

Supplementary Figure 7: ***Carbon footprint profile of battery cells with NMC811 cathodes, excluding laterite ore deposits. a*** *Probability density functions and jitter plots,* ***b*** *CF drivers and* ***c*** *variance drivers for LIB cells with NMC811 cathode using the emission curve from nickel sulfate excluding laterite, i.e. Supplementary Figure 4a instead of Figure 1d. Caption comments from Figure 2 apply. Source data are provided as a Source Data file.*

Supplementary Figures 8 and 9 present the CF distributions for lithium iron phosphate (LFP) battery cells synthesized via two methods: the solid-state and hydrothermal methods. In both figures, panels a highlight the probability density distributions of CF values, showcasing distinct characteristics between the two methods.

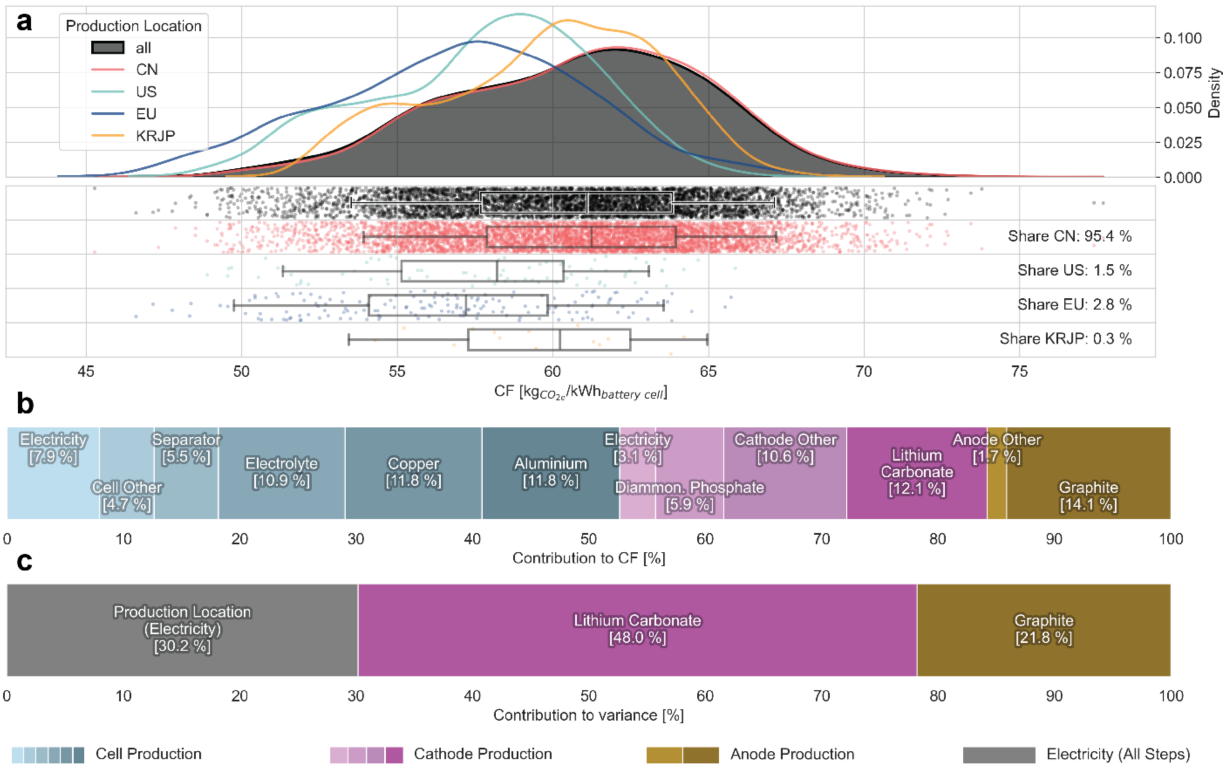

Supplementary Figure 8: ***Carbon footprint profile of battery cells with LFP cathodes synthesised via the solid-state method. a*** *Probability density functions and jitter plots,* ***b*** *CF drivers and* ***c*** *variance drivers for LIB cells with LFP cathode using the solid-state synthesis route exclusively for LFP active material. Caption comments from Figure 3 apply. Source data are provided as a Source Data file.*

The different shapes of the PDFs in Supplementary Figures 8 and 9 are due to synthesis-specific precursors, especially the iron (Fe)- and phosphorus (P)-compounds. Due to different impacts of the respective precursors, the CF and variance contribution of lithium carbonate is much greater for the hydrothermal method. This also leads to the more pronounced two-modal shape of the PDFs in Supplementary Figures 9a, owing the two plateaus of the lithium carbonate emission curve shown in Figure 1a in the main manuscript.

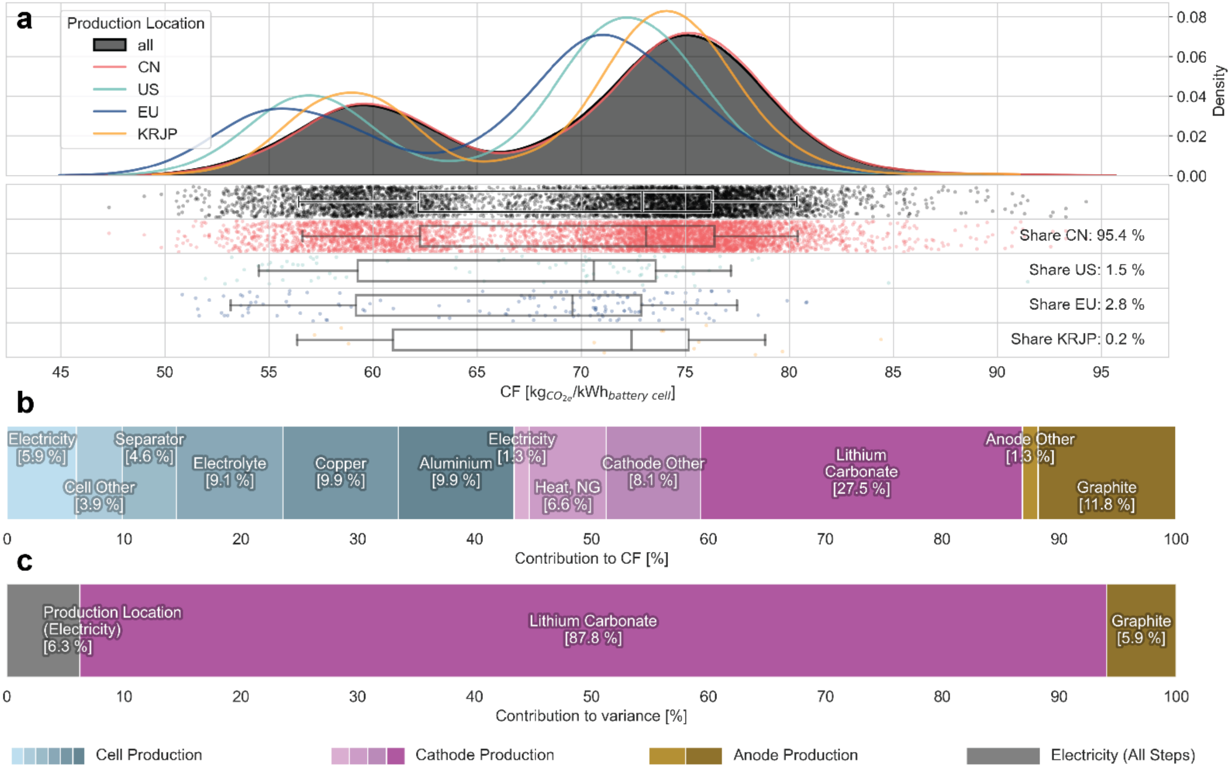

Supplementary Figure 9: ***Carbon footprint profile of battery cells with LFP cathodes synthesised via the hydrothermal method. a*** *Probability density functions and jitter plots,* ***b*** *CF drivers and* ***c*** *variance drivers for LIB cells with LFP cathode using the hydrothermal synthesis route exclusively for LFP active material. Caption comments from Figure 3 apply. Source data are provided as a Source Data file.*

Supplementary Note 4 Extensive literature review of battery material CFs

Methodology of literature review

The primary goal of this literature review was to compile a database of global warming potential (GWP) values for battery-grade raw materials used in lithium-ion batteries, including natural and synthetic graphite, nickel sulfate, cobalt sulfate and lithium carbonate. Due to the scarce availability of LCA studies for these materials, the review also encompassed studies on precursor materials, enabling the derivation of equivalent values for battery-grade substances. These precursors include Class 1 nickel, nickel hydroxide, nickel metal, cobalt hydroxide and lithium hydroxide.

To qualify for inclusion in the primary database, a publication must possess its own inventory for LCA calculations or substantially contribute to the understanding of battery-grade materials. The system boundary must be cradle-to-gate for the raw materials when examining battery cells or cathode materials. Exclusions were made for publications focusing on lab or pilot-scale processes, raw material recovery via recycling or phytomining (AKA agromining), or those solely examining energy consumption. Additionally, studies with insufficient methodological clarity or major errors were disregarded. Studies were also excluded if they did not allow for conversion to a gravimetric battery-grade raw material as the functional unit, particularly if the functional unit was expressed in terms of battery mass (kg), capacity (kWh) or cathode material mass (kg), and no disaggregation was possible.

The foundation of this database was a Scopus search, initiated by identifying and inputting relevant search keywords in various combinations for each raw material. For instance, keywords for graphite included “graphite”, “battery-grade”, “LCA”, “GWP”, and “emission”. An outline of the utilised search terms is provided below. Initially, titles were screened and promising publications were shortlisted, followed by an abstract and figure review to filter the relevant studies further. Publications that remained pertinent underwent a full-text review, with those meeting all criteria catalogued in the primary database. Publications that did not meet all criteria but were still relevant were included in a peripheral database, with a rationale for their exclusion from the primary database. Forward and backward citation tracking of the compiled studies was performed similarly, starting with title reviews, then abstracts and figures and concluding with a full-text analysis. Finally, studies in the databases were analysed using connectedpapers.com, which identified additional relevant studies not found in Scopus or in the initial citations.

In the final phase of the literature review, grey literature was searched using Google Advanced Search and the scite.ai tool, employing the same keywords from the Scopus search. The first two pages of the Google search results were examined and found to yield reports primarily from private organisations and master’s theses. Notably, the two reports covering nickel laterite production[6,18] were included in light of laterite’s importance regarding LIB CF and the lack of up-to-date peer-reviewed studies.

GWP values reported in all publications within the primary and peripheral databases were extracted. Additionally, contextual information, such as the functional unit and allocation type for the LCA, the introduction of primary data, and the study’s system boundaries, including the production process, geographical scope and life cycle inventory availability, were documented.

Moreover, our research utilised data from two key databases in life cycle assessment and environmental impact analysis: ecoinvent 3.9.1 and GREET 2022. These resources were employed to investigate the production pathways of the aforementioned raw materials from which GWP values were derived.

Results of literature review

Primary and peripheral databases for lithium, nickel, cobalt and graphite can be found in the Excel file “Literature_values.xlsx” contained in the repository. Sheets containing both qualified and peripheral studies are named “{commodity} - List”

Supplementary Table 7: Search metadata for lithium literature review.

Scopus Search Key	# results	# results after title review	# results after abstract review	# results after text review
TITLE-ABS-KEY (lca AND battery AND (li2co3 OR lioh OR (lithium AND carbonate OR hydroxide)))	17	11	8	Schenker et al. (2022) Kelly et al. (2021) Manjong et al. (2021) Jiang et al (2020) Ambrose and Kendall (2019) Jiao et al (2020) Stamp et al. (2012)
TITLE-ABS-KEY (lca AND production OR mining AND li2co3 OR lioh OR (lithium AND carbonate OR hydroxide))	18	10	8	Schenker et al. (2022) Kelly et al. (2021) Manjong et al. (2021) Jiang et al (2020) Ambrose and Kendall (2019) Jiao et al (2020) Stamp et al. (2012)
TITLE-ABS-KEY (lithium AND lib AND gwp AND production)	5	3	3	0
TITLE-ABS-KEY (lithium AND (carbonate OR hydroxide) AND (lca OR lci OR "life cycle assessment" OR gwp) AND battery AND NOT recycling)	13	11	8	Schenker et al. (2022) Chordia et al. (2022) Kelly et al. (2021) Jiang et al (2020) Ambrose and Kendall (2019) Stamp et al. (2012)
TITLE-ABS-KEY (lithium AND battery AND grade AND lca OR lci AND emission* OR gwp AND battery OR batteries OR lib)	5	4	1	Kelly et al. (2021)
TITLE-ABS-KEY (lithium AND (batter* OR lib OR lithium-ion*) AND (emission* OR co? OR ghg OR gwp) AND (lca OR life AND cycle OR inventory) AND (environmental AND impact) AND (production OR manufacture OR manufacturing) AND NOT recycling OR recycled)	95	31	17	Kelly et al. (2021) Dai et al. (2019)
TITLE-ABS-KEY (lithium AND battery OR batteries AND mining OR refining OR production AND brine OR spodumene AND emission* OR lca OR lci)	24	12	9	Khakmardan et al. (2023) Schenker et al. (2022) Kelly et al. (2021) Manjong et al. (2021) Jiang et al (2020) Telsnig et al. (2017) Stamp et al. (2012)
TITLE-ABS-KEY (lca AND (gwp OR emission* OR ghg OR co2) AND battery AND (li2co3 OR lioh OR (lithium AND (carbonate OR hydroxide))) AND (brine OR ore OR spodumene) AND (production OR manufacture OR mining))	3	2	2	Kelly et al. (2021) Manjong et al. (2021)
TITLE-ABS-KEY (lca OR (gwp OR emission* OR ghg OR co2) AND battery AND (li2co3 OR lioh OR (lithium AND (carbonate OR hydroxide))) AND (spodumene OR ore OR brine) AND (production OR manufacture OR mining))	17	11	7	Schenker et al. (2022) Kelly et al. (2021) Manjong et al. (2021) Jiang et al (2020) Ambrose and Kendall (2019)

Scopus Search Key	# results	# results after title review	# results after abstract review	# results after text review
				Telsnig et al. (2017) Stamp et al. (2012)
TITLE-ABS-KEY (lithium AND (carbonate OR hydroxide) AND (spodumene OR ore OR brine) AND (production OR manufactur* OR mining) AND (lca OR lci) AND (battery OR batteries OR lib))	10	8	6	Schenker et al. (2022) Kelly et al. (2021) Manjong et al. (2021) Jiang et al (2020) Ambrose and Kendall (2019) Stamp et al. (2012)
TITLE-ABS-KEY (lithium AND (carbonate OR hydroxide OR metal) AND (battery AND grade OR "raw material*") AND lca OR ghg OR emission*)	69	13	9	Kelly et al. (2021) Manjong et al. (2021) Ambrose and Kendall (2019) Jiao et al (2020)
TITLE-ABS-KEY (lithium AND (carbonate OR hydroxide) AND (lca OR ghg OR emission*) AND inventory)	10	4	4	Schenker et al. (2022) Manjong et al. (2021) Jiang et al (2020) Stamp et al. (2012)
TITLE-ABS-KEY (lithium AND (lca OR lci) AND (emission* OR co2 OR ghg OR gwp) AND (mining OR refining OR upstream OR production) AND (battery OR lib OR batteries) AND NOT (recycling OR recycled OR spent))	62	20	19	Kelly et al. (2021) Dai et al. (2019)

Supplementary Table 8: Search metadata for nickel literature review.

Scopus Search Key	# results	# results after title review	# results after abstract review	# results after text review
TITLE-ABS-KEY (nickel AND sulfate AND lca)	15	4	3	Jiao et al (2020)
TITLE-ABS-KEY (nickel AND sulfate AND production AND environmental AND impacts)	57	7	3	Jiao et al (2020)
TITLE-ABS-KEY (nickel AND battery AND lca)	92	25	6	Jiao et al (2020)<br>Yin et al (2019)<br>Dai et al (2019)<br>Majeau-Bettez et al (2011)
TITLE-ABS-KEY (nickel AND battery AND grade AND lca)	2	1	1	Jiao et al (2020)
TITLE-ABS-KEY (nickel AND battery AND lci)	7	6	3	Majeau-Bettez et al (2011)
TITLE-ABS-KEY (nickel AND battery AND mining AND refining AND emission* OR lca)	5	1	1	0
TITLE-ABS-KEY (nickel AND (mining OR refining OR production) AND (emission* OR co? OR gwp)) AND (LIMIT-TO (EXACTKEYWORD , "Lithium-ion Batteries"))	70	18	5	Dai et al (2019)<br>Yin et al (2019)<br>Winjobi et al (2022)
TITLE-ABS-KEY (nickel AND (sulfate OR sulfate) AND (mining OR refining OR production) AND (emission* OR co? OR gwp)) AND (LIMIT-TO (EXACTKEYWORD , "Lithium-ion Batteries"))	2	0	1	0
TITLE-ABS-KEY (nickel AND (sulfide OR sulphide) AND (batter* OR lib OR lithium-ion*) AND (emission* OR co? OR ghg OR gwp) AND (lca OR life AND cycle))	3	0	0	0
TITLE-ABS-KEY (nickel AND (batter* OR lib OR lithium-ion*) AND (emission* OR co? OR ghg OR gwp) AND (lca OR life AND cycle OR inventory) AND (environmental AND impact))	68	27	12	Majeau-Bettez et al (2011)<br>Dai et al (2019)<br>Winjobi et al (2022)
TITLE-ABS-KEY (nickel AND sulfate OR sulfate AND dataset*)	24	1	1	0
TITLE-ABS-KEY (niso4 AND batter* AND lithium-ion OR lib)	17	3	2	Jiao et al (2020)
TITLE-ABS-KEY (niso4 AND batter* AND lithium-ion OR lib AND emission OR co? OR gwp OR lca)	2	1	1	Jiao et al (2020)
TITLE-ABS-KEY (sulfate OR sulfate AND batter* AND gwp OR emission* OR co? OR impact AND lca)	11	5	4	Jiao et al (2020)
TITLE-ABS-KEY (nickel AND sulfate OR sulfate AND cathode AND material AND gwp OR co? OR emission* OR lca)	25	4	2	Jiao et al (2020)
TITLE-ABS-KEY (nickel AND sulfate OR sulfate AND automotive AND impact* OR lca OR life OR emission* OR ghg OR co?)	14	1	0	0

Scopus Search Key	# results	# results after title review	# results after abstract review	# results after text review
TITLE-ABS-KEY ("nickel sulfate" OR "nickel sulfate" AND lca OR "life-cycle" OR "life cycle" AND gwp OR emission? OR co? OR "environment* impact" OR energy AND batter* OR lib OR "lithium ion" OR "lithium-ion" OR "cathode material")	8	6	4	Jiao et al (2020)
TITLE-ABS-KEY (nickel AND "class 1" OR "nickel metal" AND lca)	25	9	5	Wei et al (2020)<br>Jiao et al (2020) Majeau-Bettez et al (2011)
"nickel class I" OR "nickel metal" AND production AND "environmental impact?" OR lca AND (LIMIT-TO (EXACTKEYWORD , "Lithium-ion Batteries"))	218	43	18	Winjobi et al (2022) Jiao et al (2020)<br>Yin et al (2019)<br>Dai et al (2019) Majeau-Bettez et al (2011)
nickel AND "class I" OR "class 1" OR "nickel metal" AND emission? OR impact OR lca OR lci OR inventory OR gwp OR co? OR ghg AND (LIMIT-TO (EXACTKEYWORD , "Lithium-ion Batteries")) AND (EXCLUDE (EXACTKEYWORD , "Recycling")) AND (LIMIT-TO (SUBJAREA , "ENVI") OR LIMIT-TO (SUBJAREA , "EART"))	176	28	8	Winjobi et al (2022)<br>Yin et al (2019) Majeau-Bettez et al (2011)
nickel AND "class I" OR "class 1" OR "nickel metal" AND emission? OR gwp OR co? AND lca AND (LIMIT-TO (EXACTKEYWORD , "Lithium-ion Batteries")) AND (EXCLUDE (EXACTKEYWORD , "Recycling"))	100	35	10	Winjobi et al (2022) Jiao et al (2020)<br>Yin et al (2019)<br>Dai et al (2019) Majeau-Bettez et al (2011)
TITLE-ABS-KEY (nickel AND "class I" OR metal AND batter* AND lca)	49	19	7	Jiao et al (2020) Majeau-Bettez et al (2011)
TITLE-ABS-KEY (nickel AND "class I" OR metal AND batter* AND lci)	3	2	1	Majeau-Bettez et al (2011)
TITLE-ABS-KEY (nickel AND "class I" OR metal AND batter* AND mining OR refining OR production AND emission? OR ghg OR gwp)	63	15	8	Winjobi et al (2022)
TITLE-ABS-KEY (nickel AND "class I" OR metal AND batter* OR lib OR lithium-ion AND emission? OR co? OR ghg OR gwp AND lca OR "life cycle" OR "life-cycle")	46	14	10	Winjobi et al (2022) Majeau-Bettez et al (2011)
TITLE-ABS-KEY (nickel AND "class I" OR metal AND batter* OR lib OR lithium-ion AND emission? OR co? OR ghg OR gwp AND inventory OR "environmental impact")	46	15	9	Winjobi et al (2022) Majeau-Bettez et al (2011)
TITLE-ABS-KEY (nickel AND "class I" OR metal AND dataset AND lithium-ion OR batter* OR lib)	14	1	0	0
TITLE-ABS-KEY (nickel AND "class I" OR "class 1" OR metal AND batter* AND lithium-ion OR lib AND emission OR co? OR gwp OR lca) AND (EXCLUDE (EXACTKEYWORD , "Recycling")) AND	31	3	2	Majeau-Bettez et al (2011)

Scopus Search Key	# results	# results after title review	# results after abstract review	# results after text review
(LIMIT-TO (EXACTKEYWORD , "Nickel")) AND (LIMIT-TO (SUBJAREA , "ENER") OR LIMIT-TO (SUBJAREA , "ENVI"))				
TITLE-ABS-KEY (nickel AND "class I" OR "class 1" OR metal AND batter* AND lithium-ion OR lib AND emission OR co? OR gwp OR lca OR impact) AND (LIMIT-TO (SUBJAREA , "ENER") OR LIMIT-TO (SUBJAREA , "ENVI")) AND (LIMIT-TO (EXACTKEYWORD , "Nickel") OR EXCLUDE (EXACTKEYWORD , "Recycling"))	42	3	2	Majeau-Bettez et al (2011)
TITLE-ABS-KEY (nickel AND "class I" OR "class 1" OR metal AND cathode AND material AND gwp OR co? OR emission* OR lca) AND (LIMIT-TO (EXACTKEYWORD , "Lithium-ion Batteries")) AND (EXCLUDE (EXACTKEYWORD , "Recycling")) AND (LIMIT-TO (SUBJAREA , "ENER") OR LIMIT-TO (SUBJAREA , "ENVI"))	36	5	2	0
TITLE-ABS-KEY (nickel AND "class 1" OR "class I" OR metal AND automotive AND impact* OR lca OR life OR emission* OR ghg OR co?) AND (EXCLUDE (EXACTKEYWORD , "Recycling") OR LIMIT-TO (EXACTKEYWORD , "Nickel"))	60	2	0	0
TITLE-ABS-KEY (nickel AND "class 1" OR "class I" OR metal AND lca OR life OR "life-cycle" OR "life cycle" AND gwp OR emission? OR ghg OR co? OR "environmental impact" AND batter* OR lib OR "lithium ion" OR "lithium-ion" AND cathode OR "cathode material")	43	13	9	Winjobi et al (2022) Jiao et al (2020)

Supplementary Table 9: Search metadata for cobalt literature review.

Scopus Search Key	# results	# results after title review	# results after abstract review	# results after text review
TITLE-ABS-KEY (cobalt AND sulfate OR sulfate AND lca)	11	5	4	Chordia et al (2021) Rinne et al. (2021)<br>Jiao et al (2020)
TITLE-ABS-KEY (cobalt AND (sulfate OR sulfate) AND battery AND lca)	**8**	4	4	Chordia et al (2021) Rinne et al. (2021)<br>Jiao et al (2020)
TITLE-ABS-KEY (cobalt AND production AND lca) AND NOT (recycling OR recycled)	11	2	0	0
TITLE-ABS-KEY (cobalt AND battery AND lca) AND NOT (recycling OR recycled)	8	4	3	Majeau-Bettez et al. (2011)
TITLE-ABS-KEY (cobalt AND battery AND grade AND (lca OR lci))	4	3	2	Rinne et al. (2021)<br>Jiao et al (2020)
TITLE-ABS-KEY ("Life Cycle Assessment" AND "cobalt" AND "battery-grade")	2	2	2	Rinne et al. (2021)
TITLE-ABS-KEY (cobalt AND battery AND mining AND refining AND emission* OR lca)	5	2	1	Zhang et al. (2021)
TITLE-ABS-KEY (*cobalt* AND (*mining* OR *refining* OR *production*) AND (*emission** OR *co?* OR *gwp*)) AND (LIMIT-TO (EXACTKEYWORD , *"Lithium-ion Batteries"*))	88	15	10	Winjobi et al (2022)<br>Dai et al. (2019)
TITLE-ABS-KEY (*"cobalt sulfate"* OR *"cobalt sulfate"* AND *lca* OR *"life-cycle"* OR *"life cycle"* AND *gwp* OR *emission?* OR *co?* OR *"environment* impact"* OR *energy* AND *batter** OR *lib* OR *"lithium ion"* OR *"lithium-ion"* OR *"cathode material"*)	7	5	3	Chordia et al (2021) Rinne et al. (2021) Zhang et al. (2021)
TITLE-ABS-KEY (*cobalt* AND *"battery grade"* OR *"sulfate"* OR *"sulfate"* OR *"metal"* AND *"production"* OR *"mining"* AND *"life cycle"* AND NOT *"recycling"*)	43	16	6	Chordia et al (2021) Rinne et al. (2021) Zhang et al. (2021)<br>Jiao et al (2020)
TITLE-ABS-KEY (cobalt AND lca OR lci OR "life cycle assessment" OR gwp AND battery AND NOT recycling)	67	20	14	Winjobi et al (2022) Accardo et al. (2021) Chordia et al (2021) Rinne et al. (2021) Zhang et al. (2021) Kelly et al. (2020)

Scopus Search Key	# results	# results after title review	# results after abstract review	# results after text review
				Jiao et al (2020) Dai et al. (2019) Majeau-Bettez et al. (2011)
TITLE-ABS-KEY (cobalt AND (sulfate OR sulfate OR chemical OR chemicals OR metal OR "battery grade") AND lca AND NOT recycling)	54	16	7	Chordia et al (2021) Jiao et al (2020) Majeau-Bettez et al. (2011)
TITLE-ABS-KEY (cobalt AND (chemicals OR metal OR powder) AND (production OR mining) AND (lca OR lci OR "impact assessment" OR inventory) AND NOT (recycling OR recycled))	129	6	3	Rinne et al. (2021) Zhang et al. (2021) Jiao et al (2020)
TITLE-ABS-KEY (cobalt AND battery OR batteries AND mining OR refining AND emission* OR lca OR lci)	35	10	5	Chordia et al (2021) Zhang et al. (2021) Kelly et al. (2020) Jiao et al (2020)
TITLE-ABS-KEY (cobalt AND (batter* OR lib OR lithium-ion*) AND (emission* OR co? OR ghg OR gwp) AND (lca OR life AND cycle OR inventory) AND (environmental AND impact))	52	12	10	Winjobi et al (2022) Rinne et al. (2021) Chordia et al (2021) Zhang et al. (2021) Kelly et al. (2020) Dai et al. (2019) Majeau-Bettez et al. (2011)

Supplementary Table 10: Search metadata for graphite literature review.

Scopus Search Key	# results	# results after title review	# results after abstract review	# results after text review
TITLE-ABS-KEY (graphite AND battery-grade AND lca)	4	4	3	Surovsteva et al (2022) Engels et al (2022a)
TITLE-ABS-KEY (graphite AND battery AND lca)	27	10	5	Surovsteva et al (2022) Engels et al (2022a) Gao et al (2018)
TITLE-ABS-KEY (graphite AND battery AND lci)	1	1	1	0
TITLE-ABS-KEY (graphite AND battery AND mining OR refining OR production AND lca)	15	8	5	Surovsteva et al (2022) Engels et al (2022a) Gao et al (2018)
TITLE-ABS-KEY (graphite AND battery AND gwp AND lca)	2	2	1	Engels et al (2022a)
TITLE-ABS-KEY (graphite AND battery AND gwp AND lca OR (life AND cycle))	3	2	1	Engels et al (2022a)
TITLE-ABS-KEY (graphite AND (mining OR refining OR production) AND (emission* OR co? OR gwp)) AND (LIMIT-TO (EXACTKEYWORD , "Lithium-ion Batteries"))	35	8	4	Engels et al (2022a)
TITLE-ABS-KEY (graphite AND (batter* OR lib OR lithium-ion*) AND (emission* OR co? OR ghg OR gwp) AND (lca OR life AND cycle))	60	5	3	Engels et al (2022a) Gao et al (2018)
TITLE-ABS-KEY (graphite AND (lca OR life*) AND (emission* OR co? OR ghg OR gwp)) AND (LIMIT-TO (EXACTKEYWORD , "Lithium-ion Batteries"))	81	9	5	Engels et al (2022a)
TITLE-ABS-KEY (graphite AND (lca OR life*) AND (emission* OR co? OR ghg OR gwp) AND (mining OR refining OR upstream))	19	2	2	Engels et al (2022a) Gao et al (2018)
TITLE-ABS-KEY (graphite AND (lca OR life*) AND (emission* OR co? OR ghg OR gwp OR energy) AND (mining OR refining OR upstream OR production)) AND (LIMIT-TO (EXACTKEYWORD , "Lithium-ion Batteries")) AND (EXCLUDE (EXACTKEYWORD , "Recycling"))	51	8	4	Surovsteva et al (2022) Engels et al (2022a)
TITLE-ABS-KEY (graphite AND (primary AND data) AND (lca OR life*))	27	2	2	Engels et al (2022a)
TITLE-ABS-KEY (natural AND graphite AND lca)	12	6	5	Engels et al (2022a) Zhang et al (2018) Surovtseva et al (2022) Gao et al (2018)
TITLE-ABS-KEY (natural AND graphite AND lca OR (life AND cycle)) AND (LIMIT-TO (EXACTKEYWORD , "Lithium-ion Batteries"))	42	4	2	Engels et al (2022a) Surovtseva et al (2022)

Scopus Search Key	# results	# results after title review	# results after abstract review	# results after text review
TITLE-ABS-KEY (natural AND graphite AND primary AND data)	22	1	1	Engels et al (2022a)
TITLE-ABS-KEY (synthetic AND graphite AND lca)	4	2	1	Surovtseva et al (2022)
TITLE-ABS-KEY (synthetic AND graphite AND lca OR (life AND cycle))	51	6	3	Surovtseva et al (2022)
TITLE-ABS-KEY (graphite AND anode AND environmental AND impact)	66	16	4	Engels et al (2022a) Surovtseva et al (2022)
TITLE-ABS-KEY (graphite AND carbon AND footprint AND batter*)	17	1	0	0
TITLE-ABS-KEY (graphite AND (carbon AND footprint OR lca OR gwp) AND automotive)	8	1	1	0

Supplementary References

1. S&P Global. S&P Capital IQ Pro: Mine Economics Cost Curve. (2024).

2. S&P Capital IQ Pro. *S&P Mine Economics Methodology*. (2023).

3. BloombergNEF. *Prices, Tariffs & Auctions Interactive Dataset*. (2024).

4. IPCC. *Aggregate Information on Greenhouse Gas Emissions by Sources and Removals by Sinks for Parties Included in Annex I to the Convention*. https://unfccc.int/sites/default/files/resource/AGI_2022_Final.pdf (2022).

5. Minviro. *Nickel's Carbon Challenge – Understanding the Relationship between Nickel Source and Carbon Intensity*. https://www.minviro.com/resources/guides/nickels-carbon-challenge (2023).

6. VDA. *PRODUCT CARBON FOOTPRINT OF NICKEL SULFATE HEXAHYDRATE PRODUCTION*. https://www.vda.de/dam/jcr:e508b237-ecfc-49ed-b9f5-c52e2a9fa658/VDA_Nickel_Sulfate_Hexahydrate_LCA_Report_2023.pdf (2023).

7. Daly, T. *Tsingshan Starts Producing EV Battery Raw Material Nickel Matte in Indonesia*. https://www.mining.com/web/tsingshan-starts-producing-ev-battery-raw-material-nickel-matte-in-indonesia/ (2021).

8. Sherritt. *Does Matte Matter - Is Nickel Pig Iron the Answer to EV Battery Demand?* https://s2.q4cdn.com/343762060/files/doc_downloads/2021/Does-Matte-Matter-Sept-2021.pdf (2021).

9. McKinsey&Company. *How Clean Can the Nickel Industry Become?* https://www.mckinsey.com/industries/metals-and-mining/our-insights/how-clean-can-the-nickel-industry-become (2020).

10. USGS. *Commodity Statistics and Information*. https://www.usgs.gov/centers/national-minerals-information-center/commodity-statistics-and-information (2024).

11. BGS. *World Mineral Statistics Data*. https://www2.bgs.ac.uk/mineralsuk/statistics/wms.cfc?method=searchWMS (2024).

12. DERA. *DERA Rohstoffinformationen: Rohstoffrisikobewertung – Graphit*. https://www.deutsche-rohstoffagentur.de/DE/Gemeinsames/Produkte/Downloads/DERA_Rohstoffinformationen/rohstoffinformationen-51.pdf?__blob=publicationFile&v=4 (2021).

13. Scott, D. Multivariate Density Estimation: Theory, Practice, and Visualization. in *Kernel Density Estimators* 125–193 (John Wiley & Sons, Inc., 1992).

14. Mutel, C. Brightway: An open source framework for Life Cycle Assessment. *J. Open Source Softw.* **2**, 236 (2017).

15. Ecoinvent. *Ecoinvent Database 3.9.1*. https://ecoinvent.org/database/ (2022).

16. Peiseler, L. Repository for publication “Carbon Footprint Distributions of Lithium-Ion Batteries and Their Materials.” (2024) doi:10.5281/zenodo.13936832.

17. Hsiang, S. *et al.* Estimating economic damage from climate change in the United States. *Science* **356**, 1362–1369 (2017).

18. Transport&Environment. *Paving the Way to Cleaner Nickel*. https://www.transportenvironment.org/wp-content/uploads/2023/10/2023_10_Briefing_Paving_way_cleaner_nickel-1.pdf (2023).
